# Effects of *Lactiplantibacillus plantarum* 19-2 on immunomodulatory function and gut microbiota in mice

**DOI:** 10.3389/fmicb.2022.926756

**Published:** 2022-08-04

**Authors:** Xiaoran Wang, Jilang Tang, Shixia Zhang, Nuannuan Zhang

**Affiliations:** ^1^College of Veterinary Medicine, Hebei Agricultural University, Baoding, China; ^2^College of Veterinary Medicine, Northeast Agricultural University, Harbin, China

**Keywords:** *Lactiplantibacillus plantarum*, immunomodulatory, immune globulin, cytokine, intestinal microbiota

## Abstract

This study aims to evaluate the effects of *Lactiplantibacillus plantarum* 19-2 (*L. plantarum* 19-2) on mice treated with the alkylating agent cyclophosphamide (CTX). Our findings show that *L. plantarum* 19-2 restored the spleen and thymus index and the number of white blood cells and lymphocytes% in CTX treated mice. Serum immunoglobulin levels in CTX-treated mice were increased by *L. plantarum* 19-2. In addition, as compared to the model group, *L. plantarum* 19-2 upregulated the content of SIgA, while *L. plantarum* 19-2 regulates the mRNA and protein expression levels of GATA-3, T-bet, IFN-γ, and IL-4 in small intestinal tissues, which adjusted mucosal barriers, structural status, and the balance of Helper T-cell 1 and Helper T-cell 2. *Lactiplantibacillus plantarum* 19-2 regulated the distribution of intestinal flora in mice, promoting the growth of *Bacteroides* and *Proteobacteria*. In addition, *L. plantarum* 19-2 inhibited the growth of several harmful bacteria, including *Actinobacteria* and *Firmicutes*.

## Introduction

Immunosuppression is a condition that inhibits the immune response caused by the damage to the body’s immune system. It will lead to the decline of the body’s ability to defend, recognize, and eliminate foreign antigens (pathogenic microorganisms, etc.) and self-antigens. It is challenging to maintain the body’s physiological balance, resulting in condition (Shini et al., 2010; Yu et al., 2014). Immunosuppressive diseases are pretty common in the veterinary clinical, which seriously endanger animal health. In addition to the immunosuppression caused by conditions, immunosuppression caused by drugs has attracted more and more attention (Huemer, 2015). In animal clinical, glucocorticoids, antigenic insecticides, sulfa antibiotics, chloramphenicol, thiamphenicol, and florfenicol can reduce immune function and can increase the risk of infection (Huemer, 2015). For example, the use of high doses of corticosteroids in the treatment of steroid-responsive meningitis-arteritis can cause severe adverse reactions and immunosuppression in animals (Giraud et al., 2021). Biedermann et al. (2016) reported that 8.1% of the dogs were euthanized due to poor response, numerous relapses, or primary corticosteroid-related side effects. Therefore, the rational use of immune enhancers to prevent and treat immunosuppressive diseases is essential.

Probiotics are living microorganisms beneficial to the body. The use of an appropriate amount will have a positive impact on the health of the host (Żółkiewicz et al., 2020). Probiotics can improve the functional state of the body by antagonizing pathogenic bacteria, promoting nutrition, and improving metabolism (Kang and Im, 2015). It can also stimulate the maturation of the immune system (Kim et al., 2021). It mainly regulates intestinal inflammation and host immune response by promoting immune cell differentiation and multiplication, releasing interleukins, tumour necrosis factor, and interferon, and transforming growth factor and other related cytokines (Galdeano et al., 2007; Azad et al., 2018). At the same time, probiotics help to maintain the homeostasis of the gut microbiota, protect the intestinal mucosal barrier, protect the host from pathogenic microorganisms, improve the body’s defense function, and enhance immunity (Falony et al., 2006; Bischoff and Kramer, 2007). However, different probiotics show different immunomodulatory abilities. *Lactiplantibacillus plantarum* (*L. plantarum*) has been reported in many studies that validate its immunomodulatory function. For example, *L. plantarum* KLDS1.0318 protects the normal immune part of the mice intestine, possibly by modulating the Th1/Th2 balance (Meng et al., 2019). In addition, the previously studied Nanometric *L. plantarum* nF1 restores normal immune function in cyclophosphamide (CTX)-induced immunosuppressed mice (Choi et al., 2018).

*Lactiplantibacillus plantarum* 19-2 is a newly discovered probiotic isolated from dog feces in our laboratory. We screened this bacterium based on the fact that it exhibited various biological activities *in vitro*. To further understand the role and mechanism of *L. plantarum* 19-2 on host immunomodulatory functions. Therefore, this experiment investigated the effect of *L. plantarum* 19-2 on the immunomodulatory function of CTX-induced immunosuppressed mice. It is hoped that a theoretical basis can be laid for exploring *Lactiplantibacillus plantarum* as an animal immune-enhancing. It provides a valuable reference for the exploration of the immune enhancing effect of probiotics on animals.

## Materials and methods

### Chemicals

Cyclophosphamide was purchased from Shanghai yuan ye Bio-Technology Co., Ltd. (Shanghai, China). *Lactiplantibacillus rhamnosus* GG (LGG) purchased from Shenzhen Yibaishun Technology Co., Ltd. (Shenzhen, China). Man-Rogosa-Sharpe medium (MRS) and SDS-PAGE Gel preparation kit were purchased from Beijing Solarbio Science & Technology Co., Ltd. (Beijing, China). The ELISA kits were bought from Beijing winter song Boye Biotechnology Co. Ltd. (Beijing, China). PrimeScript™ RT Master Mix (Perfect Real Time) was purchased from Takara Bio Co., Ltd. (Beijing, China). TB Green Premix Ex Tap II was purchased from Takara Bio Co., Ltd. (Beijing, China). TransZol Up plus RNA kit was purchased from TransGen Biotech Co., Ltd. (Beijing, China). HRP-conjugated goat antirabbit IgG secondary antibodies were purchased from Beijing Zhong Shan Goldenbridge Biotechnology Co., Ltd. (Beijing, China). Bicinchonininc acid (BCA) assay kit was purchased from Nanjing Jiancheng Bioengineering Institute (Nanjing, China). Enhanced Chemiluminescence Detection kit was purchased from New Cell & Molecuiar Biotech Co., Ltd. (Suzhou, China). Anti-T-bet, anti-GATA-3 and anti-IFN-γ antibodies were purchased from Shenyang Wanlei Biotechnology Co., Ltd. (Shenyang, China). Anti-bate-Actin and anti-IL-4 antibodies were purchased from Beijing Bioss Biotechnology Co., Ltd. (Beijing, China). All other reagents used were of analytical grade and purchased from Tianjin Fuyu Fine Chemical Co., Ltd. (Tianjin, China).

### Preparation of bacterial strain

In pre-experiments, approximate concentrations of surviving bacteria were assessed by plate viable counting, with *L. plantarum* 19-2 and LGG inoculated with MRS broth at 2% v/v, sealed with sealant to form a non-strict anaerobic environment, and incubated in a constant temperature incubator at 37°C for 24 h. Strains were incubated under these conditions at concentrations up to 1 × 10^10^ colony forming units (CFU)/ml. Then, after incubation under the above conditions, the bacteria were collected by centrifugation (3,000 × g, 5 min, 4°C), rinsed with saline three times, and the supernatant removed. The collected bacteria were diluted in sterile saline, and the concentrations of *L. Plantarum* 19-2 were adjusted to 1 × 10^10^, 1 × 10^9^, and 1 × 10^8^ CFU/ml, respectively. Similarly, the concentration of LGG was adjusted to 1 × 10^9^ CFU/ml.

### Animal and experimental design

**Figure 1:**
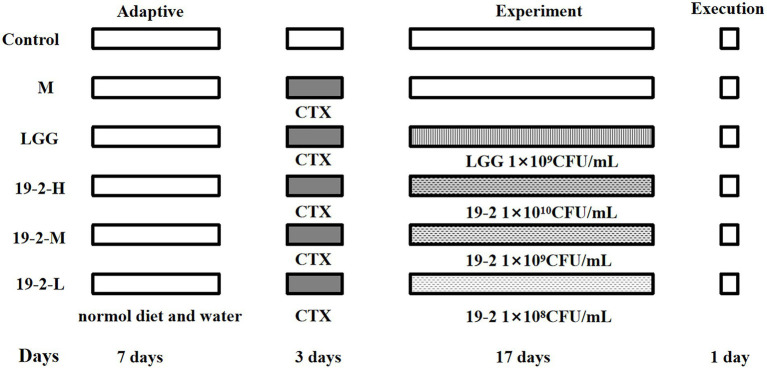
Experimental design of this study. Control group: intraperitoneal injection of saline, days 1–3 and oral saline, days 4–20; M group: intraperitoneal injection of cyclophosphamide, days 1–3; LGG group: fed with *Lactiplantibacillus rhamnosus* 1 × 10^9^ CFU/ml, days 4–20 and intraperitoneal injection of cyclophosphamide, days 1–3; 19-2-H group: fed with *Lactiplantibacillus plantarum* 19-2 1 × 10^10^ CFU/ml, days 4–20 and intraperitoneal injection of cyclophosphamide, days 1–3; 19-2-M group: fed with *Lactiplantibacillus plantarum* 19-2 1 × 10^9^ CFU/ml, days 4–20 and intraperitoneal injection of cyclophosphamide, days 1–3; 19-2-L group: fed with *Lactiplantibacillus plantarum* 19-2 1 × 10^8^ CFU/ml, days 4–20 and intraperitoneal injection of cyclophosphamide, days 1–3.

Fifty Specific Pathogen-Free (SPF) BALB/c female mice, 5 weeks of age, were purchased from SPF (Beijing) Biotechnology Co., Ltd. (Beijing, China). These animals are watered and fed freely and housed for 1 week under standard environmental conditions at room temperature (24 ± 1°C) before being used for the experiment. Mice were randomly divided into six groups (eight mice per group): normal control group (C), immunosuppression group (M), *L. Plantarum* 19-2 low dose group (19-2-L, 1 × 10^8^ CFU/ml), *L. Plantarum* 19-2 medium-dose group (19-2-M, 1 × 10^9^ CFU/ml), *L. Plantarum* 19-2 high dose group (19-2-H, 1 × 10^10^ CFU/ml) and *Lactiplantibacillus rhamnosus* positive control group (LGG, 1 × 10^9^ CFU/ml; Wen et al., 2014). Mice in each immunosuppressed group were injected intraperitoneally with CTX at a dose of 80 mg/kg/d for 3 days. All treatment groups were gavaged with the appropriate bacterial suspension at a dose of 10 ml/kg/d daily for 17 days. Meanwhile, mice in group M were gavaged with an equal amount of sterile saline. The C group mice were given equal amounts of sterile saline in the same way. During this period, the animals were watered and fed freely, and their daily body weights were recorded. All mice in the treatment and control groups were sacrificed on day 21. The CTX injection is considered the first day of the trial. The experimental design is shown in Figure 1. Blood collection from mice with their eyeballs removed, respectively. Collection of whole blood from mice in sodium heparin anticoagulation tubes for routine blood analysis. Harvesting of small intestine tissue and collection of colonic contents under sterile conditions for subsequent experiments. All collected samples were preserved at −80°C. In addition, all animal studies were done in compliance with the approval of the institutional animal care and use committee of Hebei Agriculture University.

### Weight gain and immune organ index

The body weight of the mice was assessed and recorded daily. Immediately after execution of the mice, the spleen and thymus were dissected, rinsed with saline, and then blotted dry on filter paper and weighed (mg). The spleen index and thymus index were then divided by the maternal body weight (g) and multiplied by 10 to obtain the thymus index and spleen index, respectively.

### Routine blood tests

The whole blood collected from mice was tested by a hematology analyzer (Myriad International, Shenzhen, China). Changes in the leukocyte subpopulations of the mice were detected and recorded, including the number of white blood cells (WBC) and the proportion of lymphocytes (Lymph%), monocytes (Mon%), and neutrophils (Gran%).

### Detection of serum concentrations of IgA, IgG, IgM, and intestinal mucosal SIgA and mRNA expression levels of GATA-3, T-bet, IL-4, and IFN-γ

Weigh 100 mg of small intestine tissue and add 900 μl of phosphate buffer (PBS). The small intestine tissue was well homogenized and centrifuged for 20 min (3,000 × *g*/min) to collect the supernatant of the small intestine tissue. The expression levels of cytokines SIgA were measured by ELISA kits based on the instructions. The contents of IgG, IgM, and IgA in mice serum were determined by ELISA kits based on the instructions.

The mRNA expression of GATA-3, T-bet, IL-4, and IFN-γ was detected by RT-qPCR. First, total RNA was extracted from small intestinal tissue using the TransZol Up plus RNA kit. Then, use PrimeScript™ RT Master Mix (perfect real time) according to the manufacturer’s instructions to reverse transcribes RNA into cDNA. The relative expression of the target genes was measured using the TB Green Premix Ex Tap II and the QuantStudio™ 7 Flex Real-Time PCR System (Applied Biosystems, CA, United States). Based on the correction of the housekeeping gene Glyceraldehyde-3-Phosphate Dehydrogenase (GapDH), three replicate experiments set up for each sample for the target gene and the housekeeping gene. The data were calculated using the 2^–∆∆Ct^ method (Zhao et al., 2021a). The sequences of the primers (Sangou Biotech, Co., Ltd., Shanghai, China) used for RT-qPCR are shown in Table 1.

**Table 1:** Primer sequence of RT-qPCR.

Genera	Primer sequences (5′-3′)
Gapdh	For:GGCACAGTCAAGGCTGAGAATG
	Rev:ATGGTGGTGAAGACGCCAGTA
IFN-γ	For:ACTCAAGTGGCATAGATGTGGAAG
	Rev:ACTCAAGTGGCATAGATGTGGAAG
IL-4	For:FAGTTGTCATCCTGCTCTTCTTTCTC
	Rev:TTTGGCACATCCATCTCCGT
T-bet	For:AGCTAAAGCTCACCAACAACAAGG
	Rev:GAACTGCGGGGCAACCTCTA
GATA-3	For:GCCTTCGCTTGGGCTTGAT
	Rev:AACAGATGCGTACATGGACTCAAA

### Western blot analysis

An appropriate amount of protein was extracted from small intestine tissue, and the protein concentration was determined using the BCA kit, adjusting all samples to the same concentration. The proteins were separated on 10, 12, and 15% SDS-PAGE gels respectively, and then transferred to nitrocellulose membranes. The membranes were blocked with 5% bovine serum albumin prepared in Tris-buffered saline containing 0.1% Tween 20 (TBST) for 1 h at 37°C, and then incubated with rabbit polyclonal antibodies (anti-T-bet, anti-GATA-3, anti-IL-4, anti-IFN-γ, and anti-beta-actin) overnight at 4°C and the membrane was washed with TBST (3 × 15 min). The membrane was then incubated with secondary enzyme-labelled goat anti-rabbit IgG at room temperature for 1 h and the membrane was washed with TBST (3 × 15 min). Finally the target bands were displayed by ECL chemiluminescent reagent. The bands were analyzed by Image J software.

### Histopathological examination

Histopathological sections of the jejunum were prepared in the following way. In brief, the jejunal tissues of mice were fixed in 10% formalin, dehydrated, embedded, and sectioned into paraffin sections. Routine HE staining was performed, and histopathological changes in each group of the jejunum were observed using the light microscope at 40 × magnification, and the results were collected.

### 16S rRNA high-throughput sequencing of intestinal microbial

Total microbial genomic DNA was first extracted from each fecal sample using a DNA extraction kit (Tiangen Biotech Co., Ltd., Beijing, China). The V3-V4 region of the bacterial 16S rRNA gene was then amplified by PCR using the universal primers F (5′-ACTCCTACGGGAGGCAGCA-3′) and R (5′-GGACTACHV GGGTWTCTAAT-3′). Next, the PCR amplification products were purified. Finally, sequencing was performed using the construct sequencing library. The entire 16S rRNA sequencing was done by Personal Bio-technology Co., Ltd. (Shanghai, China).

### Statistical analysis

Use GraphPad Prism 8 for all statistical analyses, and the data were statistically analyzed using SPSS 20.0 software. Measures were expressed as mean ± SD, and comparative analyses between multiple groups were performed using one-way ANOVA, with differences considered statistically significant at *p* < 0.05.

## Results

### Body weight

**Figure 2:**
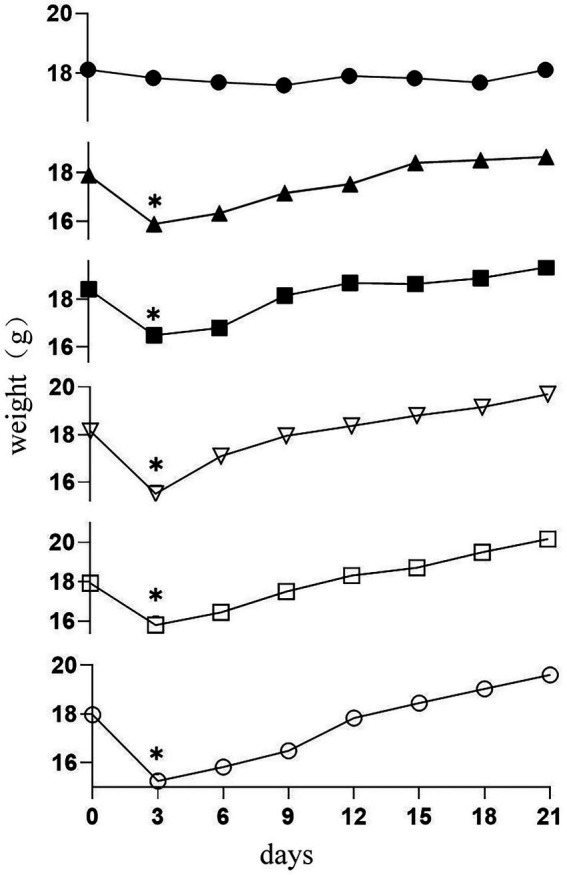
Changes in body weight in six groups of mice (●) C group; (▲) M group; (■) LGG group; (▽)19-2-H group; (□) 19-2-M group; (○) 19-2-L group. * compared with the control group, *p* < 0.05.

The body weights of the mice were tested and recorded daily. The result is shown in Figure 2; there was no difference in initial weight among the groups. Subsequently, on the third day of CTX injection in the mice, the body weight of immunosuppressed mice in five groups decreased significantly (*p* < 0.05). The results indicated that CTX reduced weight in mice. There were no significant weight gain trends among the groups throughout the remaining experimental period. Whereas the 19-2-H, 19-2-M, 19-2-L, and LGG groups gained more bodyweight compared to the M group at the end of the experiment.

### Dynamic changes in immune organ index of mice

**Figure 3:**
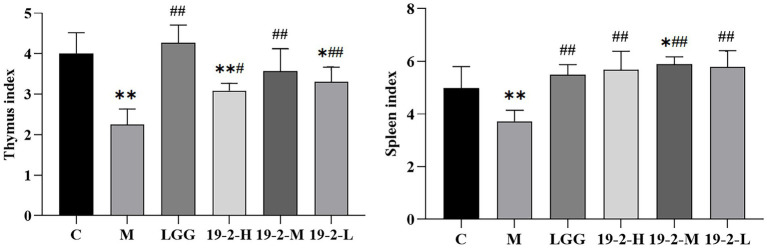
Changes in thymus index and spleen index in mice. **, * showed significant difference compared with the control group, *p* < 0.01, *p* < 0.05; ##, # represent significant difference compared with group M, *p* < 0.01, *p* < 0.05.

As shown in Figure 3, first, we noted a significant reduction in thymus and spleen indices in group M compared to group C (*p* < 0.01). However, *L. Plantarum* 19-2 and LGG treated mice in the 19-2-H, 19-2-M, 19-2-L, and LGG groups had significantly higher thymus and spleen indices compared to the M group (*p* < 0.01, *p* < 0.05). After CTX treatment, the immune organ index of mice decreased significantly, and *L. Plantarum* 19-2 and LGG promoted the recovery of immune organs of mice.

### Leukocyte subtypes in the blood of mice

**Figure 4:**
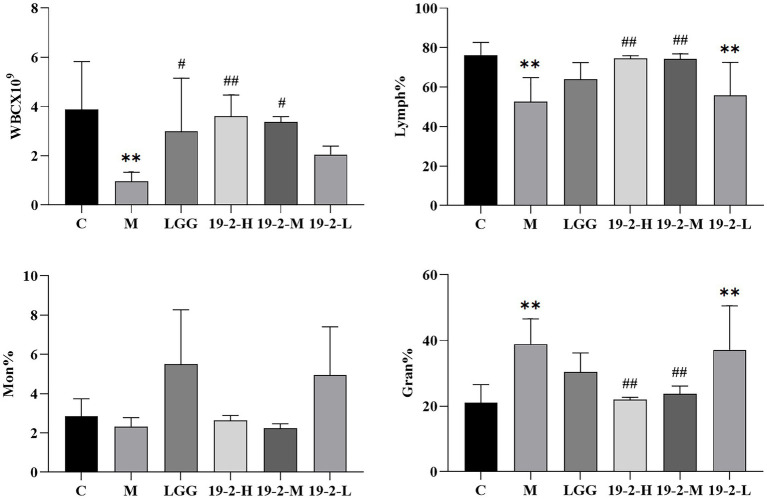
Comparison of WBC, lymphatic %, Mon%, and Gran% in blood of each group. ** showed significant difference compared with the control group, *p* < 0.01; ##, # represent significant difference compared with group M, *p* < 0.01, *p* < 0.05.

We measured and analyzed leukocyte subsets in the blood of mice, including Lymph%, WBC, Gran%, and Mon%. As shown in Figure 4, we found there was no significant difference in Mon% among all groups (*p* > 0.05). In addition, the changes in WBC and Lymph% were similar in the 19-2-H and 19-2-M groups. Compared to the normal group, WBC and Lymph% decreased significantly in the M group (*p* < 0.01), whereas this was reversed in both the 19-2-H and 19-2-M groups after treatment with *L. Plantarum* 19-2, with a significant higher in WBC and Lymph% (*p* < 0.05, *p* < 0.01). This suggests that leukocyte and lymphocyte growth is inhibited by CTX, and *L. Plantarum* 19-2 regulates this phenomenon. In contrast, Gran% was significantly higher in mice in the M and 19-2-L groups compared to the normal group, and significantly lower in mice in the 19-2-H and 19-2-M groups compared to the M group (*p* < 0.01).

### Measurement of IgM, IgG, and IgA concentrations in serum

**Figure 5:**
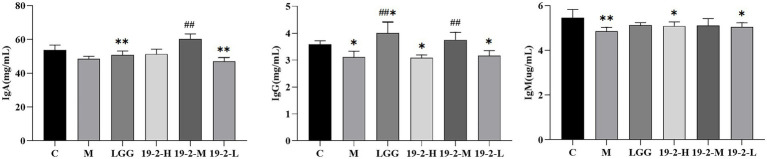
Amounts of IgA, IgG, and IgM in the blood of mice. **, * showed significant difference compared with the control group, *p* < 0.01, *p* < 0.05; ## represent significant difference compared with group M, *p* < 0.01.

The contents of IgM, IgG, and IgA in serum can reflect the immune function of the body. As shown in Figure 5, IgM, IgG, and IgA concentrations in serum were reduced to varying degrees in groups M and 19-2-L compared to group C (*p* < 0.01, *p* < 0.05). However, compared with the M group, the contents of IgA and IgG in the 19-2-M group were augmented to varying degrees (*p* < 0.01). This result suggests that CTX inhibits the synthesis of serum immunoglobulins in mice and thus affects their immune system, and LGG and *L. Plantarum* 19-2 have a restorative effect on the immune system.

### Concentration of SIgA and mRNA and protein expression levels of cytokines IL-4, IFN-γ, GATA-3, and T-bet in the intestinal mucosa

**Figure 6:**
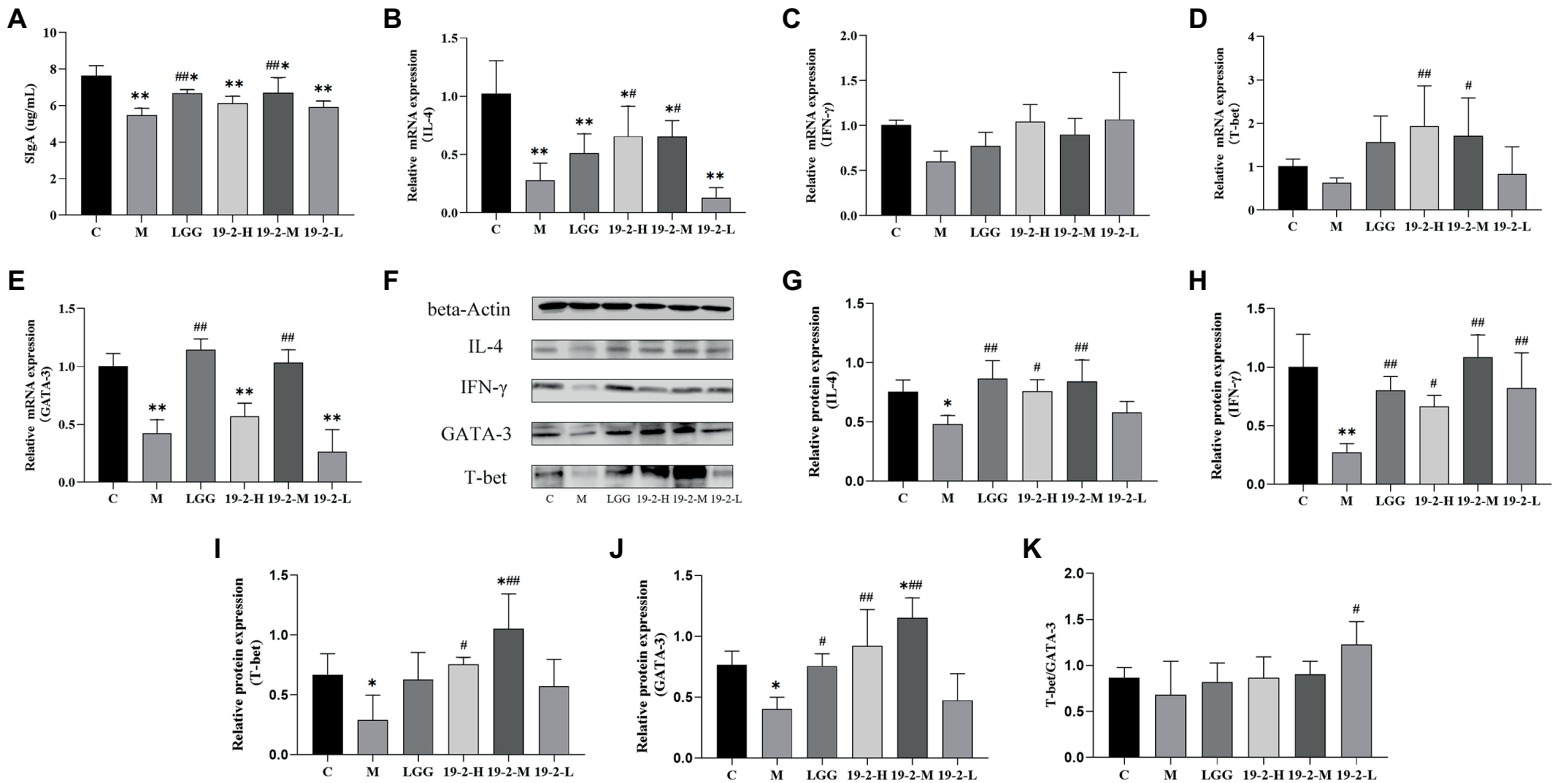
Concentration of SIgA and mRNA and protein expression levels of cytokines in the intestinal mucosa. **(A)** Concentration of SIgA in the small intestinal mucosa. **(B–E)** The mRNA expression levels of IL-4, IFN-γ, T-bet, and GATA-3. **(F)** The protein levels of IL-4, IFN-γ, T-bet, and GATA-3. **(G–J)** Values from densitometry of IL-4, IFN-γ, T-bet, and GATA-3 were normalized to the level of beta-Actin protein. **(K)** Relative intensities of T-bet/GATA-3 bands. **, * showed significant difference compared with the control group, *p* < 0.01, *p* < 0.05; ##, # represent significant difference compared with group M, *p* < 0.01, *p* < 0.05.

To assess the effect of *L. Plantarum* 19-2 on the CTX-induced immune barrier in the intestinal mucosa, we measured the concentrations of SIgA in the intestinal mucosa of mice. As shown in Figure 6A, compared with the C group, the contents of SIgA in the remaining groups varied degrees of reduction significantly (*p* < 0.05, *p* < 0.01). Interestingly, SIgA concentrations were significantly higher in the LGG and 19-2-M groups than in the M group (*p* < 0.05), suggesting that the toxic effects of CTX inhibited SIgA secretion in the small intestinal mucosa, which was ameliorated by supplementation of LGG and *L. Plantarum* 19-2 in mice.

To evaluate the mechanism of *L. Plantarum* 19-2 on CTX-induced immunosuppression, we determined the mRNA and protein expression levels of IL-4, IFN-γ, GATA-3, and T-bet in the small intestine. As shown in Figure 6B, the expression of IL-4 mRNA was significantly lower in each treatment group compared to group C (*p* < 0.05, *p* < 0.01) and CTX significantly reduced IL-4 mRNA expression. Interestingly, the expression levels of IL-4 mRNA were increased in the 19-2-H and 19-2-M groups compared with the M group (*p* < 0.05). However, the IFN-γ mRNA expression level displayed no significant difference in the four-strain treatment groups and the M group (*p* > 0.05; Figure 6C). The expression of GATA-3 and T-bet regulates Th1 and Th2 differentiation. Hence, the expression of GATA-3 and T-bet is critical in the Th1/Th2 balance. As shown in Figure 6D, the expression level of T-bet mRNA was significantly higher in the 19-2-H and 19-2-M groups compared to the M group (*p* < 0.01, *p* < 0.05). Notably, the mean expression level of T-bet mRNA expression in the M group was 0.627758, lower than that in other groups. As shown in Figure 6E, the expression level of GATA-3 mRNA in group M was significantly lower than that in group C (*p* < 0.01), but there was no significant difference in the expression of GATA-3 mRNA in groups 19-2-H and 19-2-L compared with group M. Interestingly, GATA-3 mRNA expression levels were significantly higher in the LGG and 19-2-M groups than in the M group (*p* < 0.01). These results suggest that *L. Plantarum* 19-2 regulates the mRNA expression levels of GATA-3 and T-bet.

As shown in Figures 6G–K, CTX induction could lead to the downregulated protein expression of IL-4, IFN-γ, GATA-3, and T-bet in mouse ileum (*p* < 0.01, *p* < 0.05). Treatment with *L. Plantarum* 19-2 could increase the relative expression of IL-4, IFN-γ, GATA-3, and T-bet in the ileum tissues of mice (*p* < 0.01, *p* < 0.05). The protein expression of IFN-γ was higher in all four treatment groups than in the M group (Figure 6H; *p* < 0.01, *p* < 0.05). As the results in Figures 6G,I,J show that the levels of IL-4, T-bet, and GATA-3 in 19-2-H and 19-2-M groups were higher than that of M group (*p* < 0.01, *p* < 0.05). Such effects in 19-2-M group were stronger than those of other treatment groups. To better understand the regulation of the Th1/Th2 balance by *L. Plantarum* 19-2, we calculated and analyzed the ratio of T-bet/GATA-3. Specifically, as shown in Figure 6K, the T-bet/GATA-3 ratio decreased in the M group and increased in the 19-2-H, 19-2-M, and 19-2-L groups. In the 19-2-L group, the T-bet/GATA-3 ratio increased significantly (*p* < 0.05).

### Histopathological changes in the small intestine

**Figure 7:**
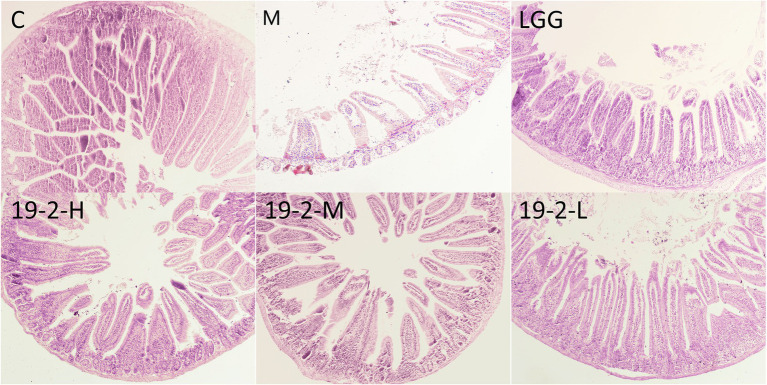
Section of jejunum under light microscope at 400 magnification.

To further evaluate the intestinal function of *L. Plantarum* in immunosuppressed mice treated with CTX, the histopathology of the jejunum was evaluated with HE staining. As shown in Figure 7, the normal jejunum sample exhibited neatly arranged villi that were slender and compact. In contrast, the jejunal samples from group M were disrupted and severely reduced in villi after CTX treatment. Prominent cell edema and vacuole bodies appeared. After *L. Plantarum* 19-2 was treated, improved the structural state of the jejunum compared with the CTX-treated sample.

### Dynamic changes of microbiota in fecal samples

The data from the above study showed that the high dose group of the strain did not show a strong improvement in the animal model of CTX inhibition, with the medium dose group showing the best results. Total DNA was extracted from the 19-2-M group which was renamed as the A19-2 group in the following experiments. Based on the high sequencing of 16S rRNA in the V3 and V4 hypervariable regions. Regarding feces microbiota, a total of 442,804 sequences, the sequence length of the samples ranged from 404 to 432 bp, were assigned to 409 OTUs on average per sample, and clustered into 58 genera and 13 phyla. The abundance of OTUs initially reflects the species richness of the samples. As shown in Table 2, OTUs were found to be significantly lower in both the M and A19-2 groups compared to the C group (*p* < 0.05, *p* < 0.01). The Good for all three groups reached over 98%, covering almost all sequences and achieving the depth of microbial analysis required for sequencing. The alpha diversity determines the richness and homogeneity of the microflora. As shown in Table 2, the number of Chao1 and Shannon indices in the M group and A19-2 group increased in contrast to the C group.

**Table 2:** OTUs and α-diversity indices of microbiota in each group.

	OTUs	Chao1	Shannon	Simpson	Goods_coverage
C	56344.7 ± 4986.3	1423.93 ± 190.81	4.72 ± 1.54	0.72 ± 0.24	0.988
M	42621.3 ± 2813.1**	3179.79 ± 635.1**	8.95 ± 0.74**	0.99 ± 0.008	0.981
A19-2	45649.3 ± 4565.0*	2822.48 ± 879.28*	7.38 ± 1.49*	0.91 ± 0.085	0.980

**, * showed significant difference compared with the control group, *p* < 0.01, *p* < 0.05.

**Figure 8:**
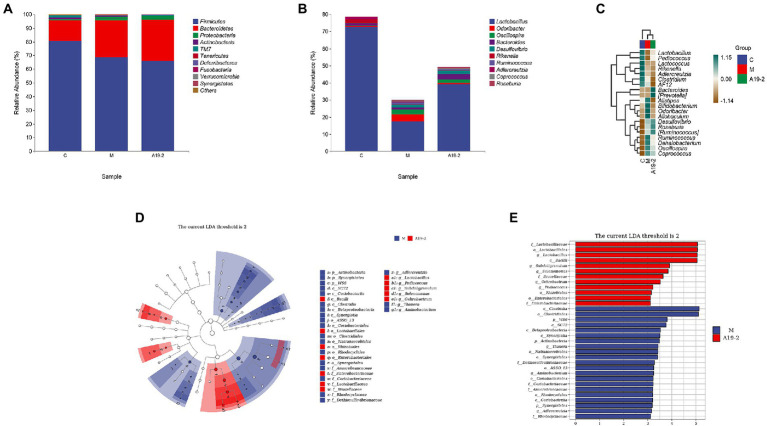
Dynamic changes of intestinal microbial taxonomic composition. **(A)** Analysis of intestinal microbial abundance at phylum level; **(B)** analysis of intestinal microbial abundance at genus level; **(C)** heat map of intestinal microbiota at genus level; **(D)** LEfSe taxonomic cladogram. Colored nodes from the outer ring to the inner ring represent the relationship between the intestinal flora from the genus level to the phylum level; and **(E)** groups with LDA scores >3.

In order to know more about the specific changes of intestinal flora, we analyzed the classification and composition of microbiota in the samples. All samples belonged to four main phyla, *Bacteroidetes*, *Firmicutes*, *Actinobacteria*, and *Proteobacteria*. Among them, *Bacteroidetes* and *Firmicutes* were the most, reaching more than 90%. The relative abundance of *Proteobacteria* and *Bacteroidetes* tended to increase with CTX intervention, while the relative abundance of *Firmicutes* and *Actinobacteria* tended to decrease. However, the use of *L. plantarum* 19-2 has made this trend even more pronounced (Figure 8A). However, this trend was exacerbated by using *L. Plantarum* 19-2. At the genus level (Figure 8B), *Lactobacillus*, *Odoribacter*, *Oscillospira*, *Bacteroides*, *Desulfovibrio*, *Rikenella*, *Ruminococcus*, *Adlercreutzia*, *Coprococcus*, and *Roseburia* were the 10 most predominant microbiota, with the *Lactobacillus*, having highest abundance. The results are shown in Figure 8C, the abundance of *Lactobacillus*, significantly reduced after CTX treatment compared to the normal control group. However, the abundance of *Lactobacillus* in group A19-2 was higher than that in group M, indicating that *L. Plantarum* 19-2 showed some improvement in the abundance of *Lactobacillus* in the intestinal microbial community in response to CTX. Interestingly, *Bacteroides* abundance was increased in the A19-2 group and decreased in the M group compared to the C group. Moreover, *Odoribacter*, *Oscillospira*, and *Ruminococcus* were raised in the M group. However, *L. Plantarum* 19-2 treatment attenuated the effects caused by CTX, and the abundance of *Odoribacter*, *Oscillospira*, and *Ruminococcus* reduced. In addition, *Rikenella* and *Adlercreutzia* decreased, but *Desulfovibrio*, *Coprococcus*, and *Roseburia* considerably raised in the M and A19-2 groups compared with the Control group.

We performed LEfSe analysis on each group, an evolutionary clustering analysis of the major microbiota to identify the critical system types of the intestinal flora. As shown in Figure 8D, *Synergistic* and *Clostridiales* were the highest in the red area, and in the cladogram, *Coriobacteriia*, *Saprospirae*, and *Bacilli* had the richest flora abundance in the purple parts, in which purple and red are represented by the C group and M group, respectively. To determine changes in key system types of *L. Plantarum* 19-2 intestinal flora in different groups of CTX-treated mice. We performed LEfSe analysis in group M and group A19-2. According to the LDA results (Figure 8E), there were six dominant microbiota in the M group, with *Clostridia* and *Clostridiales* being the main microbiota (LDA > 3.5, *p* < 0.05), and eight dominant microbiota in the A19-2 group (LDA > 3.5, *p* < 0.05), with the dominant bacterial groups adjusted to *Lactobaciliaceae*, *Lactobacillales*, *Lactobacillus*, and *Bacilli*. Thus, this result indicates that treatment with *L. Plantarum* 19-2 altered the classification of key microflora in the gut of CTX-treated mice promoting the proliferation of specific bacteria.

## Discussion

Immunosuppressive diseases are increasingly studied in animal because of the threat it poses to the health of the body. Probiotic is a general term for a group of active microorganisms that colonize the intestinal and reproductive systems of animals, producing definitive health effects and thus improving the host micro-ecological balance and exerting beneficial effects (Torres-Henderson et al., 2017). It has been shown that *Lactiplantibacillus* and *Candida Savagella* can exert potent immunomodulatory activity in animals (Oh et al., 2010; Yin et al., 2013). However, the colonization of the animal gut by specific microorganisms can be compromised by host specificity. A growing number of reports have identified the role of the intestinal flora on immune function and its regulation in immune disorders (Eslami et al., 2019; Yousefi et al., 2019). Therefore, we need to perfect our studying of the link between candidate probiotics and immune system defense to create more effective interventions through clinical and molecular testing. The objective of this study was to investigate the effect of *L. Plantarum* 19-2 on immune function in CTX-treated mice and to determine whether it is protective of immune function in mice.

The spleen and thymus are the most important immune organs of the body and are the main sites of lymphocyte differentiation, maturation, and immune effect. The thymus indices and spleen indices are also an indication of the body’s immunity and reflect the animal’s immune function (Oh et al., 2018). The results showed that the thymic and splenic indices of the four groups treated with the strains were significantly greater than those of the M group, and the splenic index could restore to near-normal levels. This indicates that *L. Plantarum* 19-2 can improve the effects caused by CTX on immune organs.

Leukocytes play a vital defense role in the organism, participating in the body’s defense response by phagocytosis of bacteria and prevention of disease. An increase or decrease in leukocyte subsets can reflect the immune function of mice. Lymphocytes are a kind of white blood cells produced by lymphoid organs. They are an essential part of the immune response function of the body (Park et al., 2019). The number of WBC and Lymph% in the *L. Plantarum* 19–2 treatment group increased gradually. However, apparent enhancements appeared after *L. Plantarum* 19-2 treatment at all two doses except the low for 19-2-L. The results suggest that *L. Plantarum* 19-2 significantly increases the number of Lymph% and WBC in immunosuppressive model mice caused by CTX and improves the immune function of mice. Gran% were substantially increase in all groups compared to the normal group, but Mon% was not significantly different. Similarly, found Gran% to be higher in the M and 19-2-L groups than those in the control group, while it was reduced in the 19-2-H and 19-2-M groups. Therefore, we propose that the increased Gran% may be due to CTX reducing immune function and causing infection, with *Lactiplantibacillus plantarum* acting as a protective agent and reducing infection.

Immunoglobulin in serum is an essential part of the body’s immune system, and its content reflects the strength of the body’s immune function to some extent. IgM, IgA, and IgG are protein molecules with antibody activity secreted by B-lymphocytes, which have functions of complement activation, antiviral, and cell phagocytosis in the body. They are usually used as important indicators to monitor the immune system function of the body (Wang et al., 2011; Long et al., 2021). The concentrations of IgG, IgM, and IgA in the serum of CTX-treated mice were significantly reduced, possibly due to the cytotoxic effect of CTX on B cells and plasma cells or inhibition of B cell differentiation, interfering with humoral immunity and thus leading to lower antibody concentrations in the blood. The results showed that CTX was effective in suppressing humoral immunity, which is consistent with previous studies (Hussein and Ahmed, 2016; Okuda et al., 2018; Zhao et al., 2021b). Compared with the model control group, the concentration of serum IgA and IgG of the mice in middle dose of *L. Plantarum* 19-2 group were increased. These results suggest that *L. Plantarum* 19-2 can enhance the humoral immunity function of the body.

The intestine is not only a digestive organ for the absorption of nutrients but also a critical defense barrier in the animal’s body (Bischoff and Kramer, 2007). Immune cells are widely distributed in the intestinal mucosa of healthy animals, and because of the combined presence of the SIgA, tight junction proteins, mucus layer, and antimicrobial factors form an efficient intestinal immune barrier. The intestinal mucosal barrier thus performs an absorptive function while maintaining a stable intestinal environment and protecting the host from harmful bacteria and viruses (Mantis et al., 2011). A large amount of SIgA produced by the plasma cells of the intrinsic mucus layer plays an essential function in the immune function of the intestine, which prevents bacterial invasion of the intestine. Furthermore, the lack of SIgA in the intestine can lead to an overgrowth of bacteria in the gut, resulting in a bacterial imbalance that disrupts the homeostasis of the intestine (Cui et al., 2020). The results showed that *L. Plantarum* 19-2 increased the content of ileal SIgA. Thus, *L. Plantarum* 19-2 plays a vital role in protecting the intestinal mucosa from the effects of CTX to prevent it from causing SIgA deficiency. This result may be since the entry of *L. Plantarum* 19-2 into the intestinal maintains the stability of the intestinal mucosal immune response and regulates the dynamic interaction between intestinal mucosal cells and these microorganisms, leading to an increase in the secretion of SIgA and thus regulating the intestinal mucosal immune system.

Th cells play an integral part in the body’s immune system by proliferating and spreading to activate immune cells. Each Th cell subpopulation regulates or assists in the immune response by recognizing different antigens and secreting signature cytokines (Yamane and Paul, 2013). Th1 cells normally regulate the cellular immune response, producing interferon INF-γ. Th2 cells produce cytokines to enhance the humoral immune response. The typical cytokine secreted by Th2, IL-4, can usually be detected early in their development (Aruga et al., 1997; Glimcher and Murphy, 2000). Under normal circumstances, cytokines secreted by Th1 and Th2 regulate each other to maintain dynamic balance (Lan et al., 2021). Compared with the C group, the IL-4 mRNA expression levels were significantly lower in all groups. Meanwhile, the differences in IFN-γ mRNA levels between the groups were not statistically significant. However, *L. plantarum* 19-2 increased the expression level of IL-4 mRNA. Our findings showed that the IFN-γ and IL-4 protein levels all significantly decreased in CTX treated model group compared to normal group, but all of them were upregulated after the treatment of *L. plantarum* 19-2. The above results suggest that *L. plantarum* 19-2 regulates the cytotoxic effect of CTX on small intestinal mucosal Th cells, mainly by regulating the production of transcription factors released by IL-4 produced by Th2 cells and stimulating the secretion of cytokines IL-4 and IFN-γ.

The transcription factors GATA-3 and T-bet promote Th cell development and are major regulators of Th2 and Th1 differentiation, respectively (Chakir et al., 2003). Th1/Th2 balance is the key to maintaining normal immune function (Xiao et al., 2012). Expression of the T-bet and GATA-3 affects the stability of Th1/Th2 homeostasis (Yates et al., 2004; Xie et al., 2015). The status of Th1/Th2 can be reflected by the T-bet/GATA-3 ratio. Previous research has reported that Lactiplantibacillus can influence the immune response effects of Th1/Th2 (Borchers et al., 2002). Therefore, we assessed the expression levels of GATA-3 and T-bet mRNA and protein in ileum tissue. The results showed that the mRNA and protein expression of GATA-3 and T-bet were decreased in group M compared with group C, indicating that CTX had an inhibitory effect on intestinal mucosal immunity in mice. After the addition of *L. plantarum* 19-2, the expression levels of T-bet mRNA and protein were significantly higher in the 19-2-H and 19-2-M groups compared with the M group. As well as protein expression levels of GATA-3 were significantly higher in the 19-2-H and 19-2-M groups. It is suggested that *L. plantarum* 19-2 has the effect of enhancing the immune function of small intestinal mucosa. However, the expression level of mRNA in the 19-2-H group was significantly lower than that of the normal group. This may be due to the fact that the mRNA was already degraded by the time the protein expression reached its peak (de Sousa Abreu et al., 2009). It has been suggested that CTX inhibits the release of Th1 cytokines, which ultimately leads to the migration of Th1 cells to Th2 cells (Finotto et al., 2002). We find that the T-bet/GATA-3 ratio was reduced in the CTX-treated group and increased after *L. plantarum* 19-2 treatment, suggesting that *L. plantarum* 19-2 may regulate CTX-induced migration of Th1/Th2 to Th1. Thus, it was shown that *L. plantarum* 19-2 could improve intestinal immune function in CTX-induced mice by regulating Th1/Th2 cells to achieve homeostasis. As the dose of *Lactobacillus plantarum* 19-2 decreased, the ratio of T-bet/GATA-3 increased, suggesting that our different *Lactobacillus* strains and doses may induce different immune responses (Tsai et al., 2012).

The use of probiotics needs to be selected and doses determined according to individual characteristics and specific needs (Gonchar et al., 2014). Our study found that the medium dose group of *L. plantarum* 19-2 had a normal effect on CTX-induced immune organ indices, blood tests, toxicity of serum immunoglobulin and SIgA concentrations, and disturbance of Th1/Th2 balance. Notably, the therapeutic effect of *L. plantarum* 19-2 did not show a dose dependence. This means that in order for *L. plantarum* 19-2 to work optimally, we need to pay attention to its dose. Wang et al. (2017) treated broilers infected with *Clostridium perfringens* different doses of *Lactiplantibacillus* and showed that low doses of *Lactiplantibacillus* improved fat metabolism and intestinal permeability in broilers. Rao et al. (2018) found a large bacterial bloom in the small intestine of patients with cerebral blur (long-term probiotic users), as well as bacterial fermentation of carbohydrates to produce D-lactic acid, which is temporarily toxic to brain cells and interferes with cognition, thinking and sense of time. When patients stopped taking probiotics for a period of time, their symptoms improved.

The outermost layer of the small intestinal epithelium consists of a large number of villi and crypts with a surface area of over 400 m^2^. In contrast, the epithelial cells are covered with a thick layer of mucus, which is rich in mucin glycoproteins, antimicrobial peptides, and SIgA, thus facilitating the efficient absorption of nutrients (Macpherson et al., 2011). This study found that CTX caused morphological abnormalities in small intestinal villus cells of jejunum in the M group. In contrast, all three groups showed significant relief of abnormal intestinal symptoms after *L. Plantarum* 19-2 treatment. This evidence indicate that *L. Plantarum* 19-2 plays an important role in maintaining the integrity of the intestinal epithelial barrier and thus protecting the first line of immune defense in mice.

The physiology of animals is influenced and regulated by trillions of microorganisms in the body. The gastrointestinal tract of animals is teeming with microbes and is known as gut microbiota (Eckburg et al., 2005). These symbiotic bacteria maintain intestinal homeostasis and enhance host immune function through communication with the host small intestinal epithelium and bacterial interactions (Suganya and Koo, 2020). At the same time, the gut microbiota is thought to shape and regulate the host immune system (Kundu et al., 2017). This is because the altered colonization of the gut by probiotics affects the survival of harmful pathogens in the gut, which then activates an immune response to clear them (Eslami et al., 2020). In addition, some strains can inhibit the growth of harmful bacteria by producing bacteriocins. For example, bacteriocins produced by *Lactiplantibacillus acidophilus* La-14 inhibit the multiplication of *Listeria monocytogenes* (Todorov et al., 2011). α-Diversity index analysis showed that the samples had good microbial community diversity and richness. There are various pathogenic bacteria in the *Actinobacteria* phylum, such as *Brachybacterium*, *Corynebacterium*, and *Arthrobacter* that often cause purulent diseases and local inflammation. Many bacteria in firmicutes are drug-resistant, including *Staphylococcus*, *Sporococcus*, and *Jeotgaliccoccus* (Zhao et al., 2021b). The abundance of Actinobacteria phylum and Firmicutes phylum in the A19-2 group was slightly lower than that in the normal group and M group. Thus, *L. Plantarum* 19-2 can protect the intestine of mice by inhibiting the multiplication of harmful bacteria in the intestine. *LactobacilliLacti* have various beneficial effects on their hosts, and *Lactobacilli* are a common measure of the effectiveness of probiotics at the genus level (Khodaei et al., 2016). The immune dysfunction induced by CTX leads to the decrease of *Bacteroides*, which may cause infection. Our results showed that *L. Plantarum* 19-2 recovered *Lactiplantibacillus* and *Bacteroides*. According to LEfSe analysis, *Clostridium* was the major microbiota in group M, and *Clostridium* is usually harmful to the host’s health. However, compared with group M, the major microbiota of A19-2 changed to lactic acid bacteria. In summary, *L. Plantarum* 19-2 can regulate the composition and structure of the intestinal microbial community of CTX-treated mice, maintain intestinal microbial homeostasis, and has the potential to regulate intestinal microbial ecology.

## Conclusion

In conclusion, this study shows that CTX-treated immunosuppressed mice gavaged with *L. Plantarum* 19-2 can improve the toxic effects induced by CTX, normalize all parameters, and improve the organism’s immunity. Moreover, it could regulate the Th1/Th2 balance, improve intestinal microbial distribution and intestinal morphology to maintain intestinal flora homeostasis and enhance immune function. Therefore, the study of *L. Plantarum* 19-2 is of great significance as it can be used as an effective immunomodulator and may be applied in the development of animal immune enhancers.

## Data availability statement

The datasets presented in this study can be found in online repositories. The names of the repository/repositories and accession number(s) can be found at: https://www.ncbi.nlm.nih.gov/, SRP362804:PRJNA813386.

## Ethics statement

The animal study was reviewed and approved by Ethics Committee of Hebei Agricultural University, China.

## Author contributions

XW and SZ participated in the design of this study. XW and JT carried out the study and collected important background information, performed the statistical analysis, and drafted the manuscript. SZ and JT performed manuscript review. NZ provided assistance for data acquisition, data analysis, and statistical analysis. All authors contributed to the article and approved the submitted version.

## Funding

This study was supported by the Science and Technology Project of Hebei Education Department (BJ2019053) and National Natural Science Foundation of China (grant number 31802250).

## Conflict of interest

The authors declare that the research was conducted in the absence of any commercial or financial relationships that could be construed as a potential conflict of interest.

## Publisher’s note

All claims expressed in this article are solely those of the authors and do not necessarily represent those of their affiliated organizations, or those of the publisher, the editors and the reviewers. Any product that may be evaluated in this article, or claim that may be made by its manufacturer, is not guaranteed or endorsed by the publisher.

## Supplementary material

The Supplementary material for this article can be found online at: https://www.frontiersin.org/articles/10.3389/fmicb. 2022.926756/full#supplementary-material
