# Supplementary figures and images for: Effects of Lactiplantibacillus plantarum 19-2 on immunomodulatory function and gut microbiota in mice
Source: Front Microbiol. 2022 Aug 4;13:926756. doi: 10.3389/fmicb.2022.926756 (PMC9386500; doi:10.3389/fmicb.2022.926756)

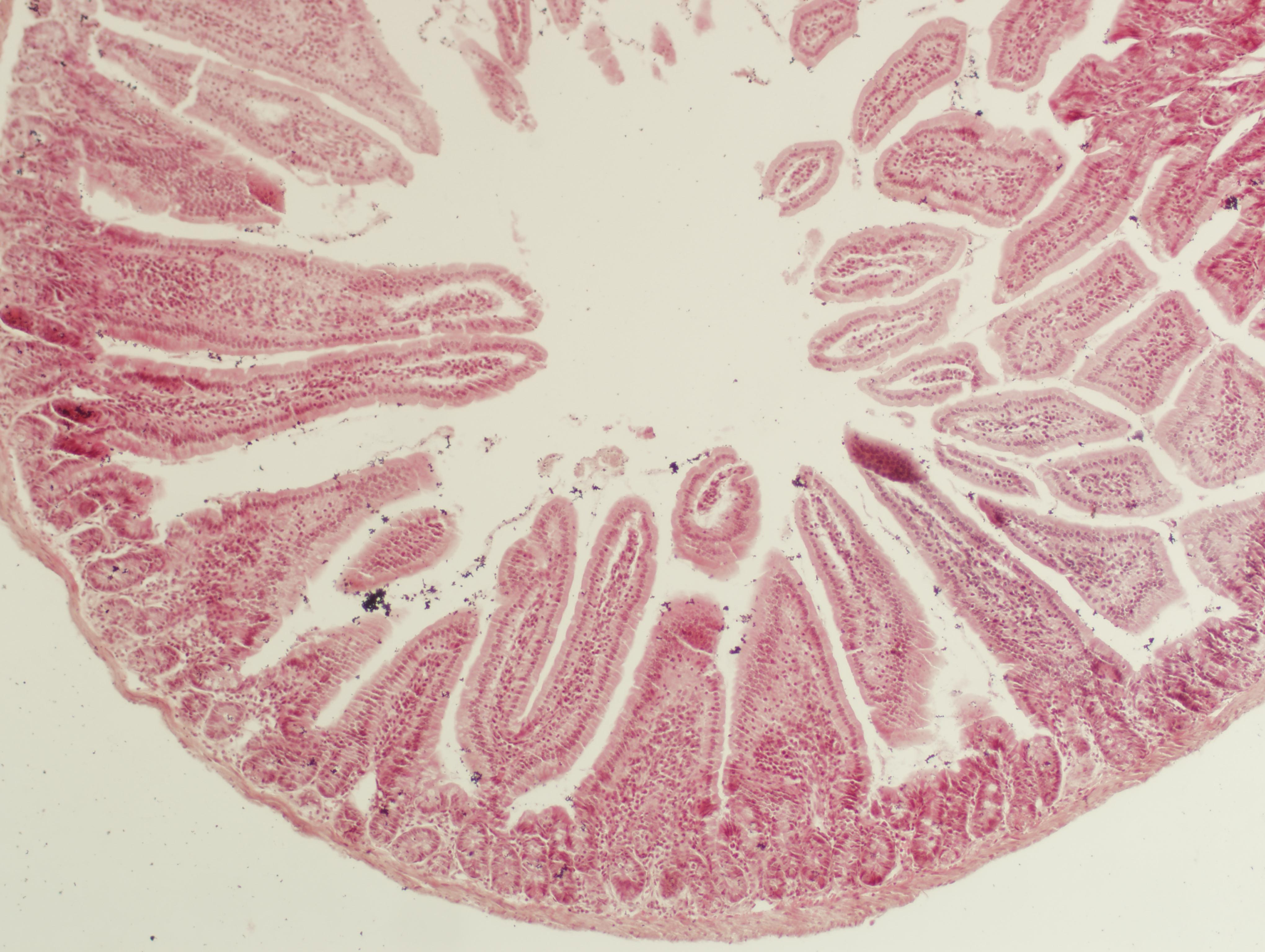

Supplement: Supplementary file 1 [file Data_Sheet_1.ZIP › Raw Data/Tissue section image/19-2-H.jpg]

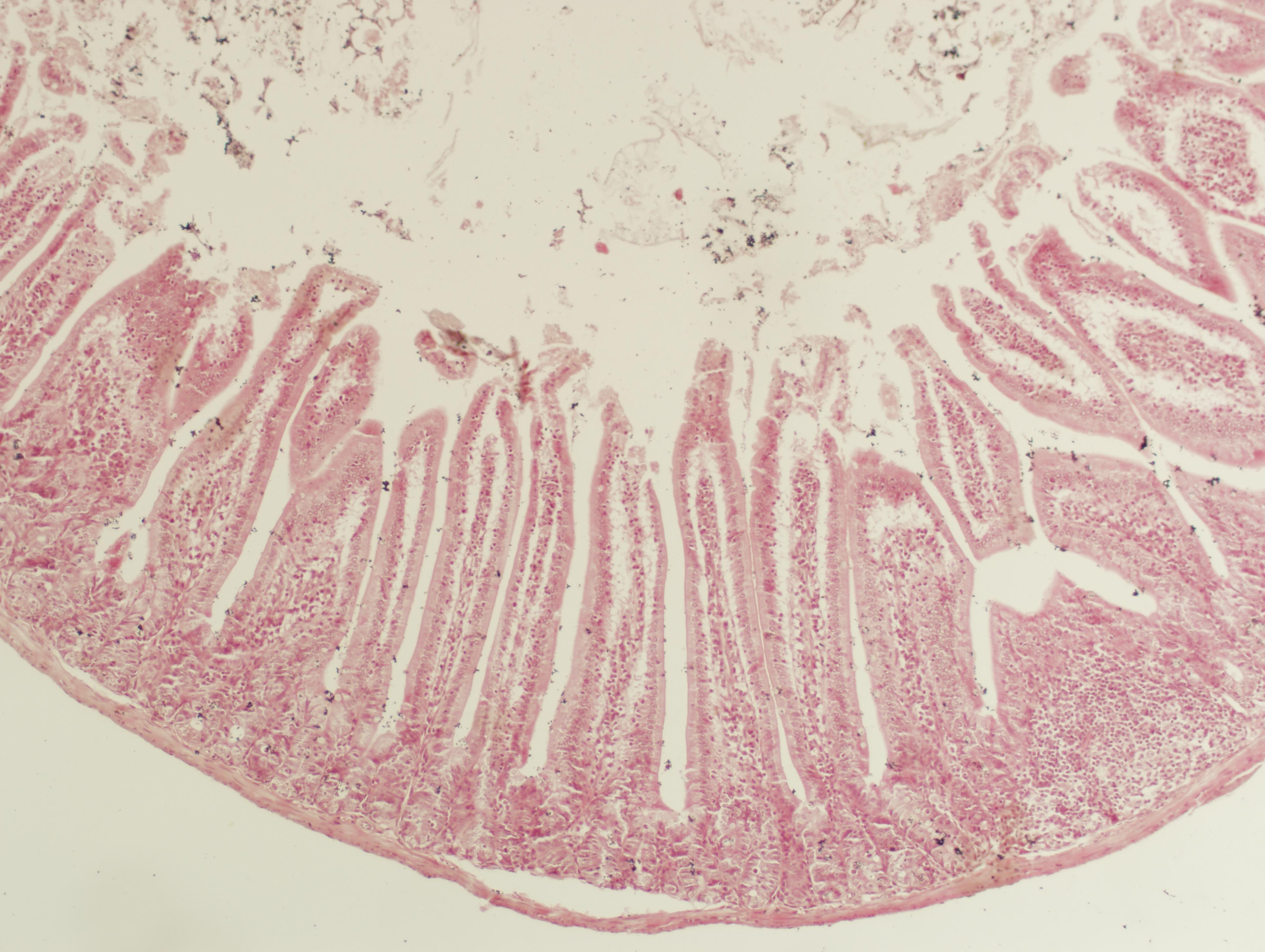

Supplement: Supplementary file 1 [file Data_Sheet_1.ZIP › Raw Data/Tissue section image/19-2-L.jpg]

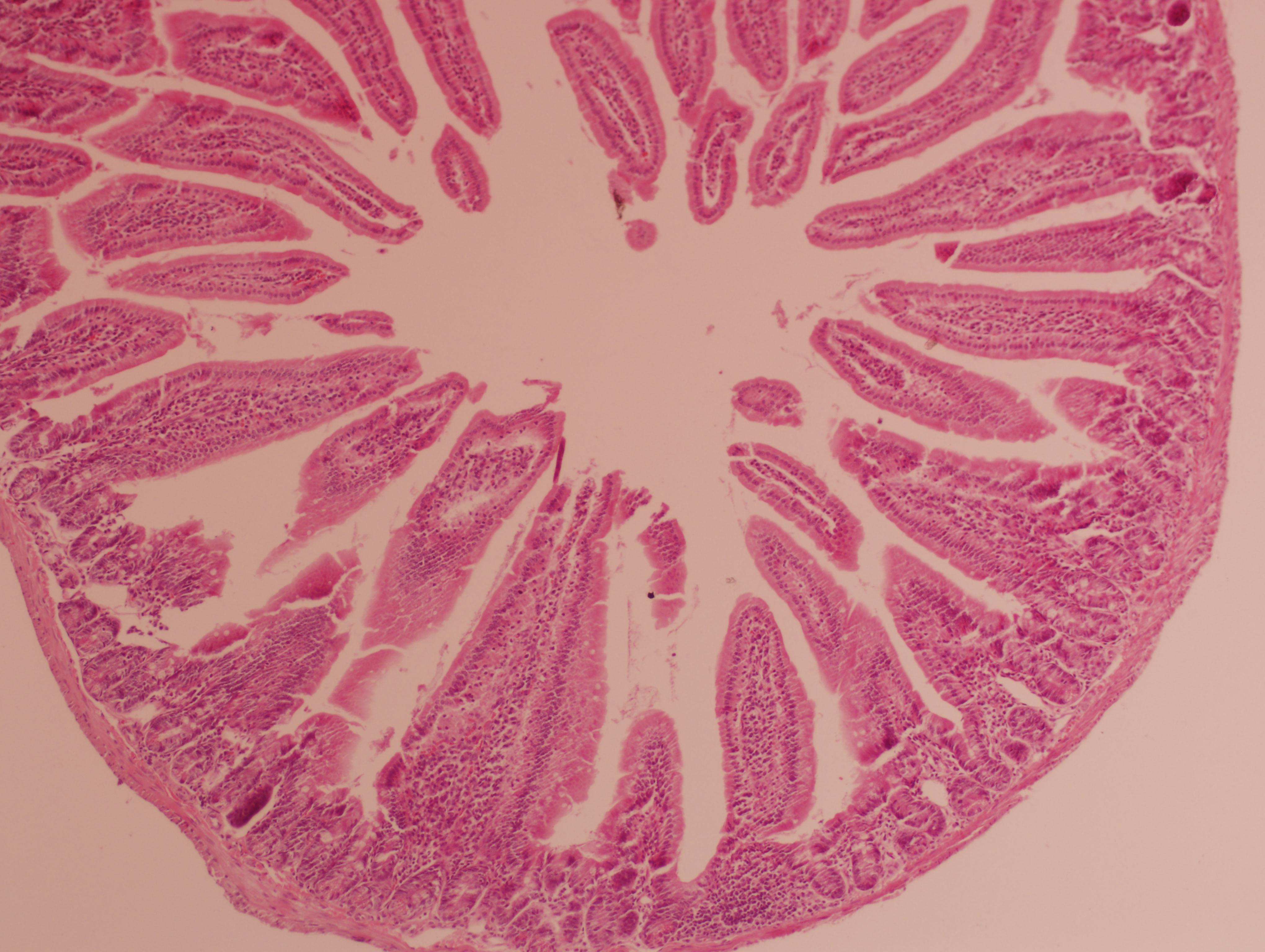

Supplement: Supplementary file 1 [file Data_Sheet_1.ZIP › Raw Data/Tissue section image/19-2-M.jpg]

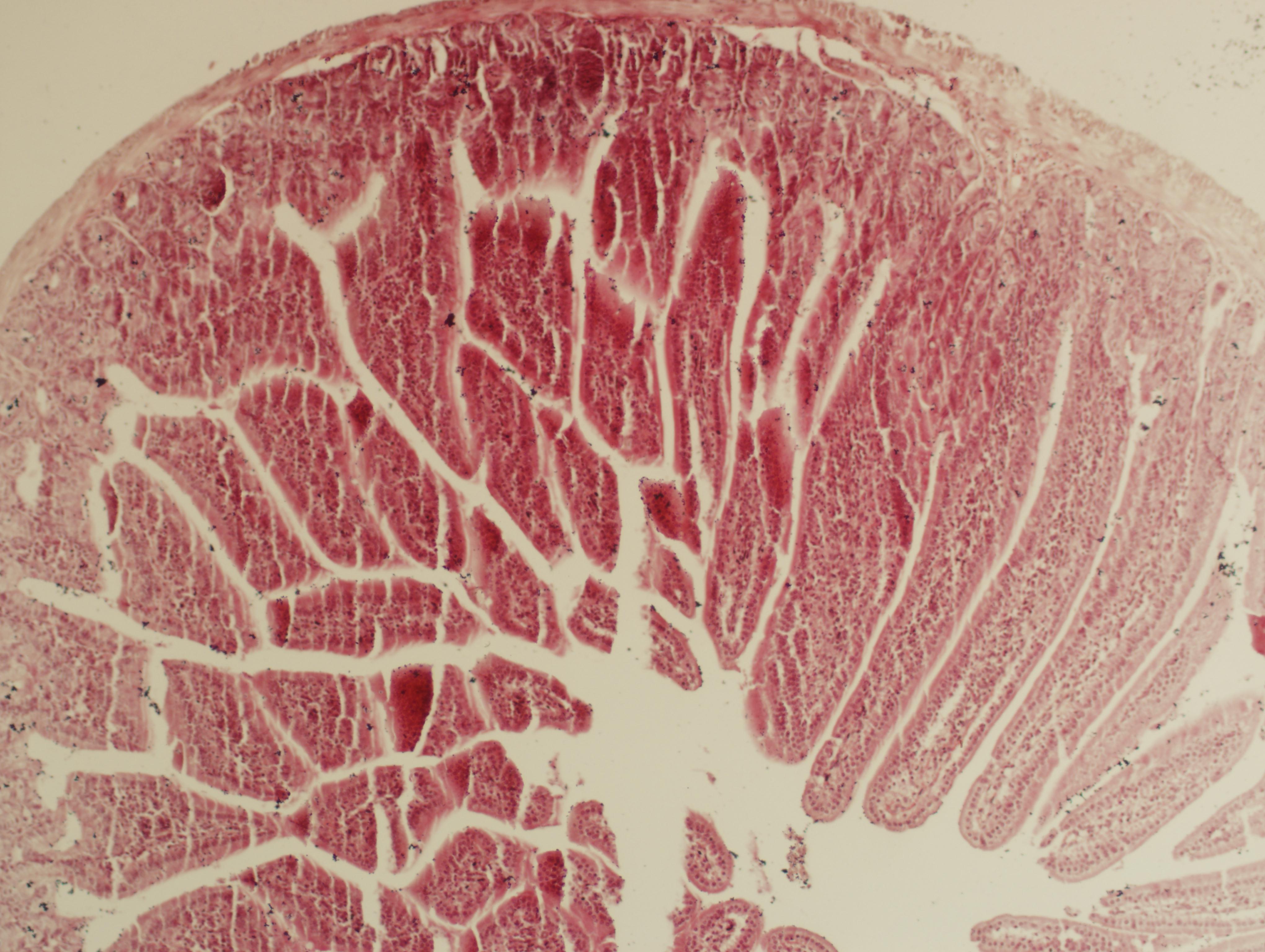

Supplement: Supplementary file 1 [file Data_Sheet_1.ZIP › Raw Data/Tissue section image/C.jpg]

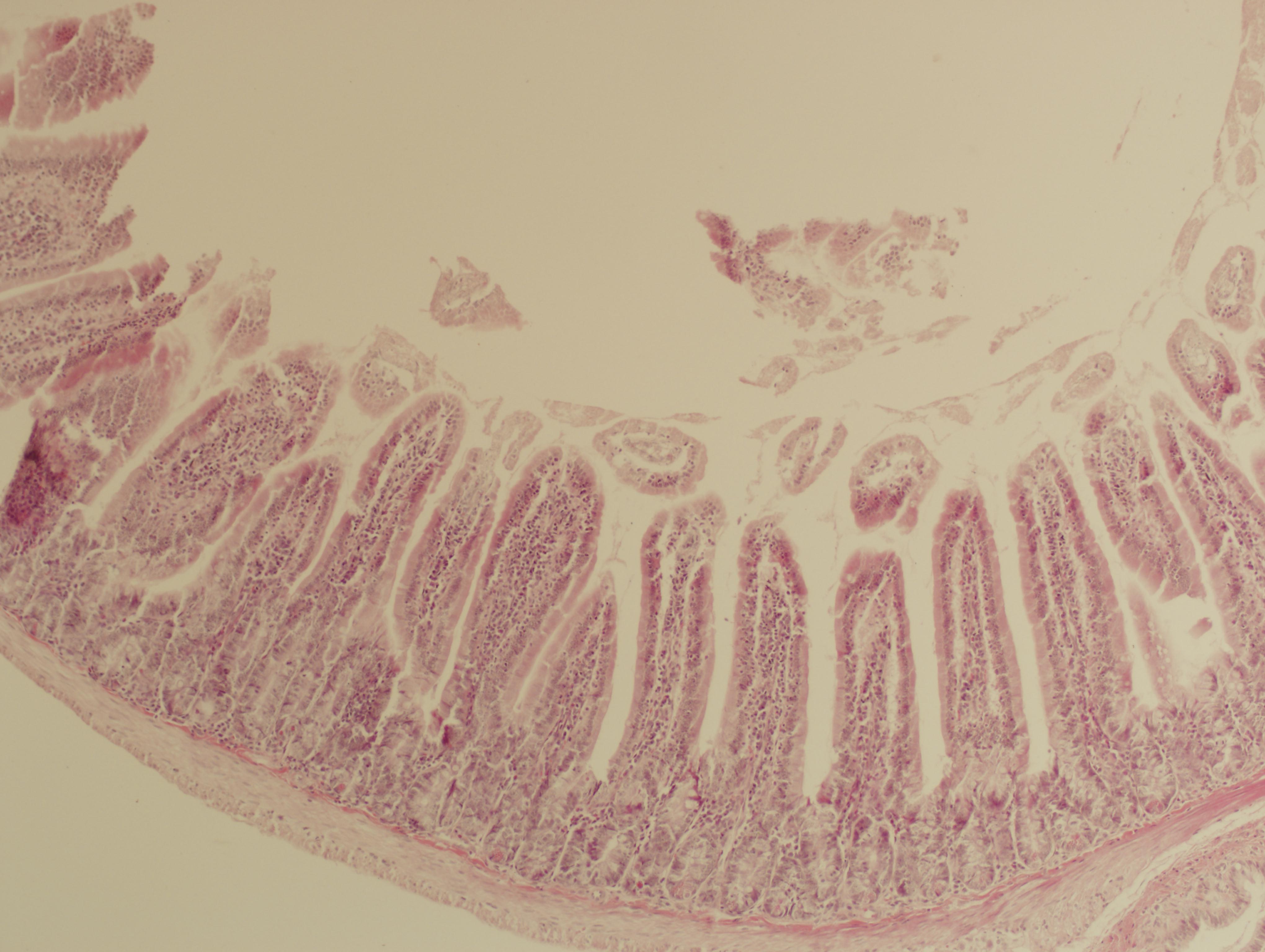

Supplement: Supplementary file 1 [file Data_Sheet_1.ZIP › Raw Data/Tissue section image/LGG.jpg]

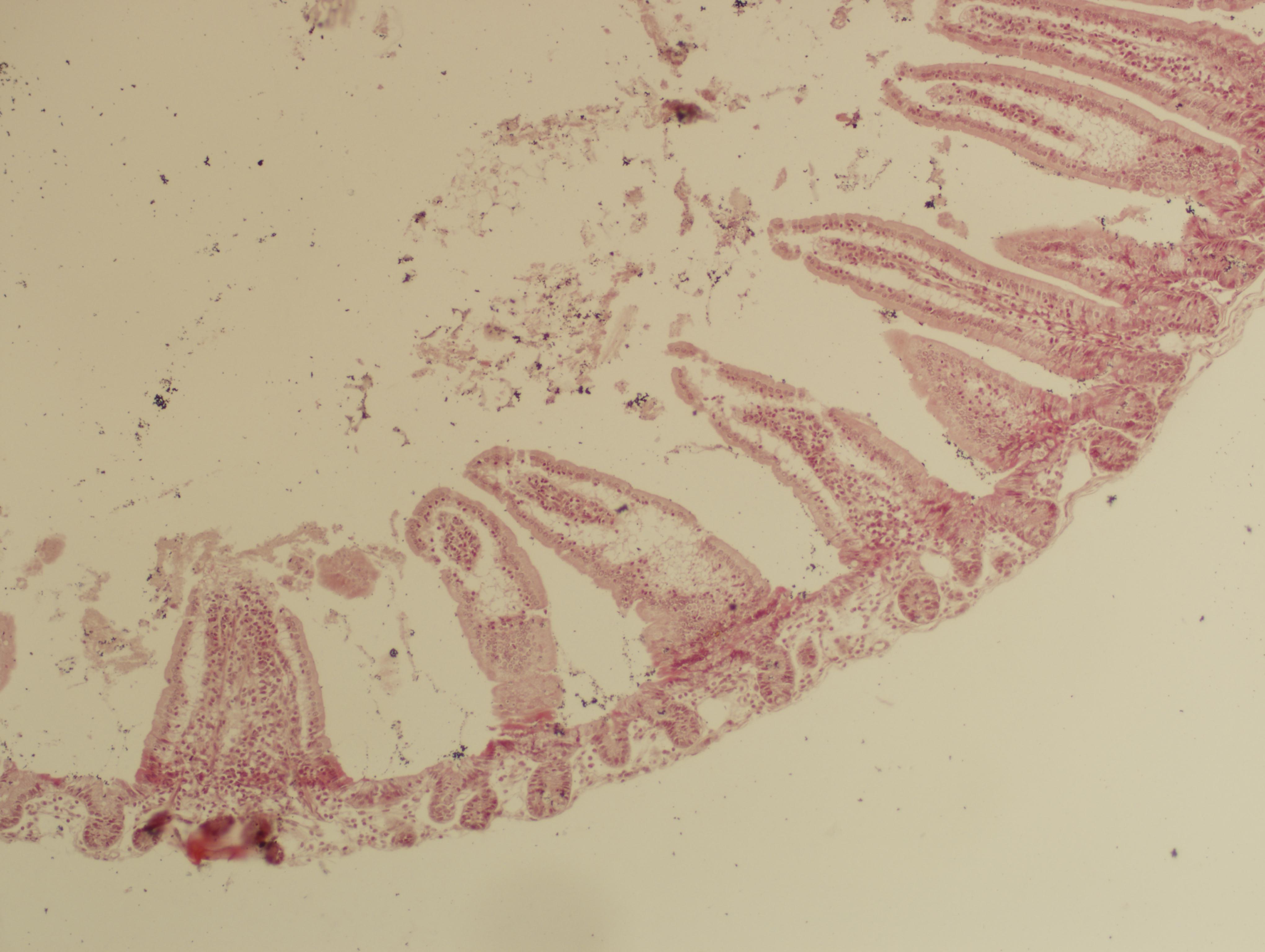

Supplement: Supplementary file 1 [file Data_Sheet_1.ZIP › Raw Data/Tissue section image/M.jpg]

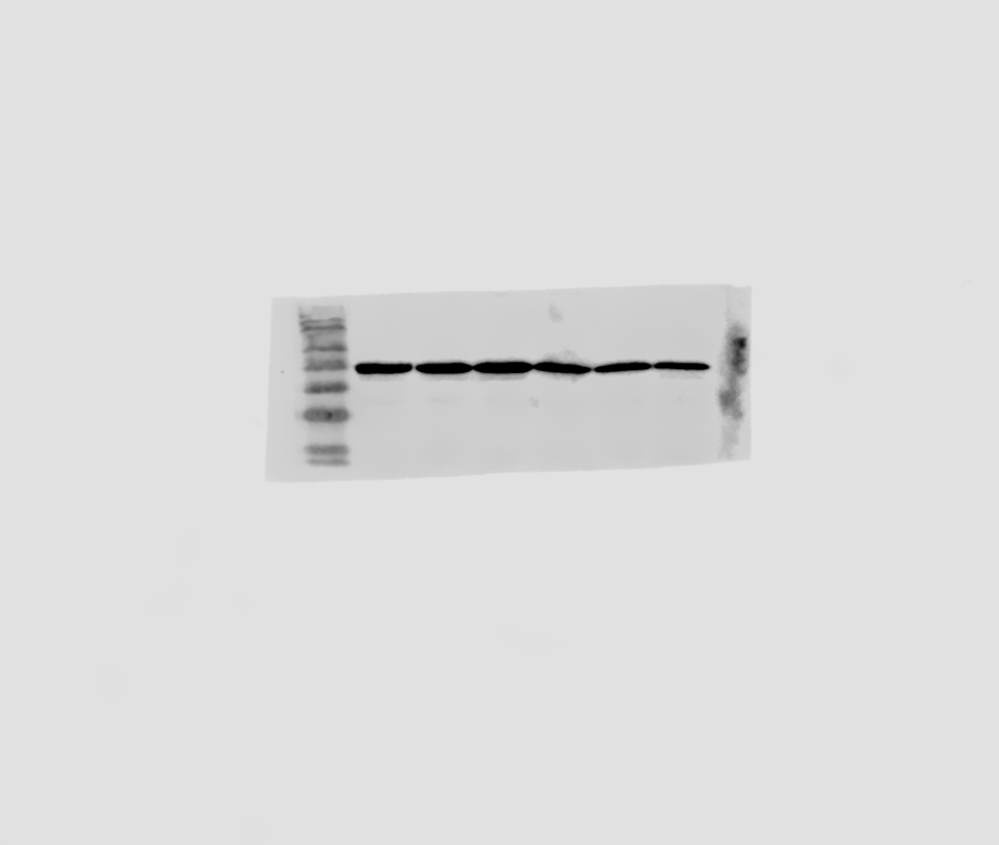

Supplement: Supplementary file 1 [file Data_Sheet_1.ZIP › Raw Data/Western Blot/bete-Actin/1.png]

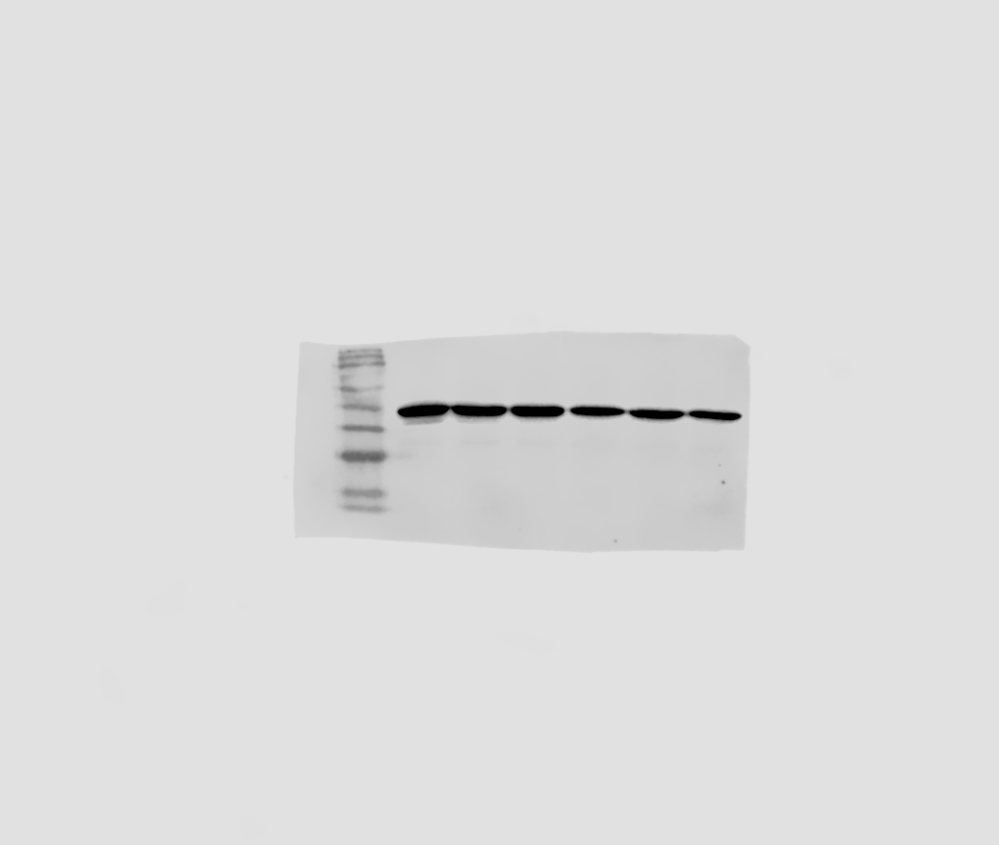

Supplement: Supplementary file 1 [file Data_Sheet_1.ZIP › Raw Data/Western Blot/bete-Actin/2.png]

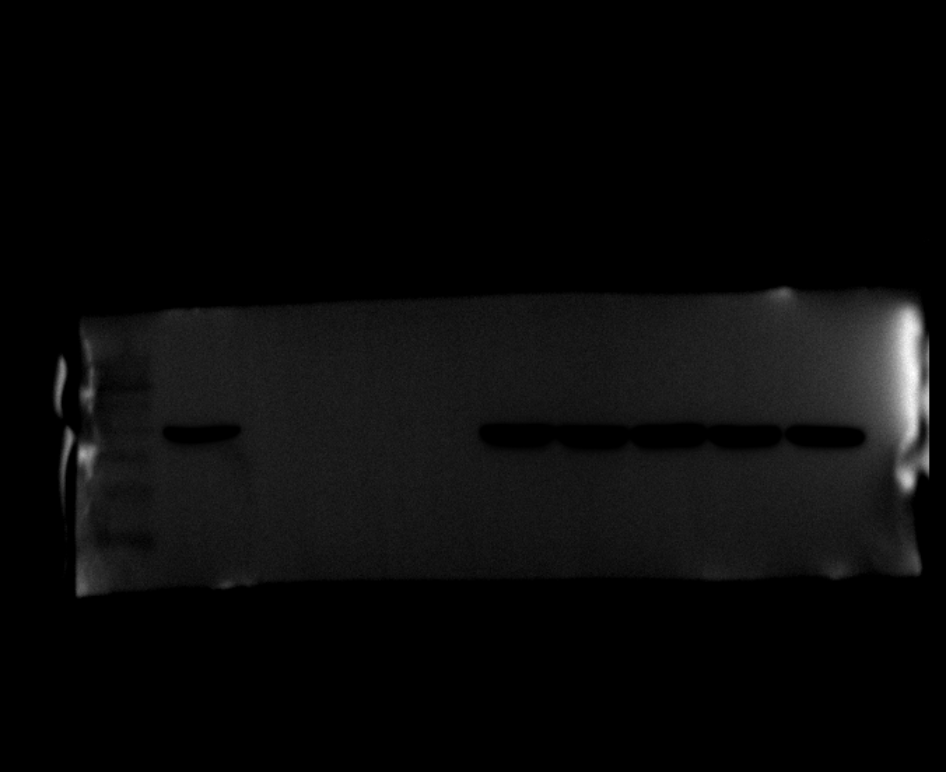

Supplement: Supplementary file 1 [file Data_Sheet_1.ZIP › Raw Data/Western Blot/bete-Actin/3-Marker.tif]

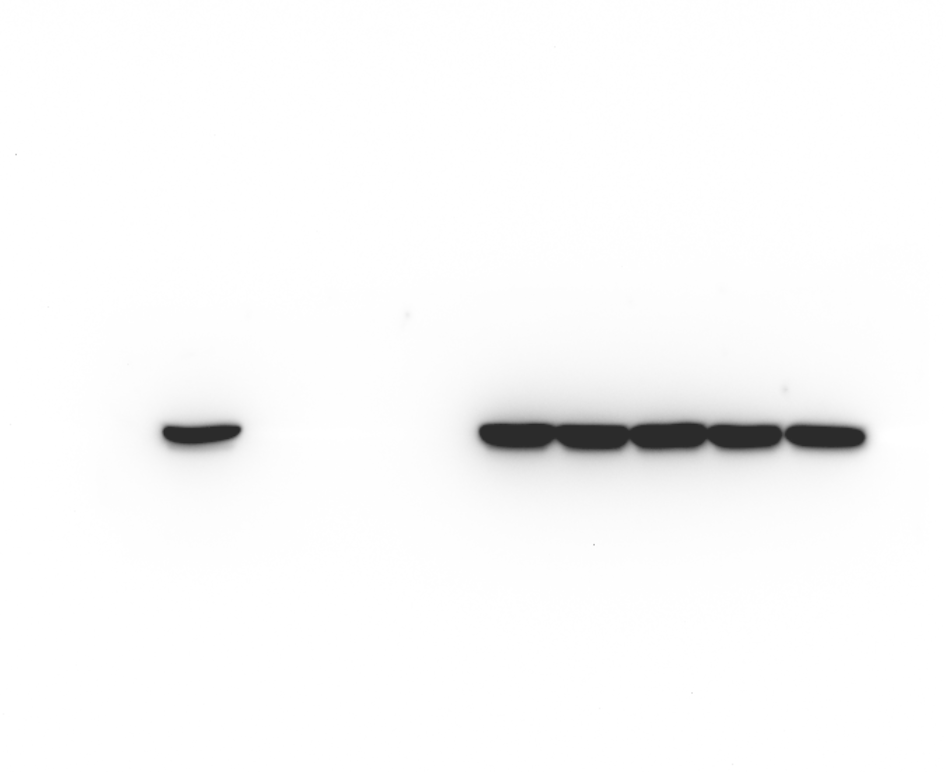

Supplement: Supplementary file 1 [file Data_Sheet_1.ZIP › Raw Data/Western Blot/bete-Actin/3.tif]

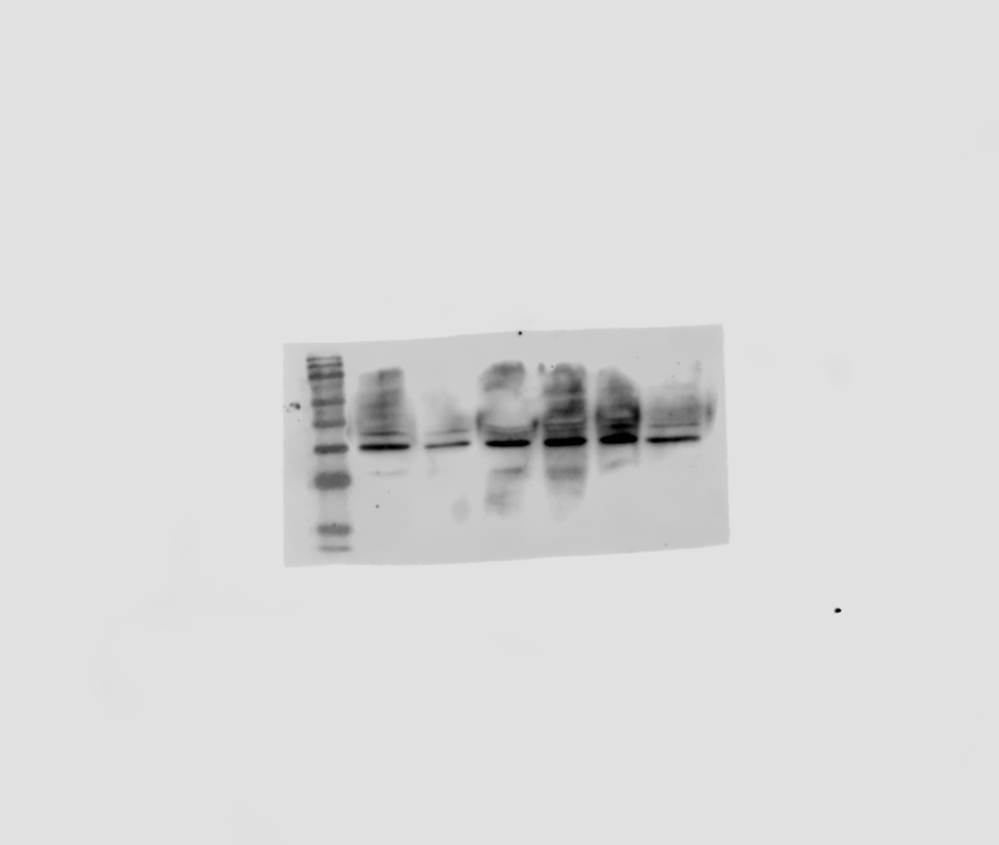

Supplement: Supplementary file 1 [file Data_Sheet_1.ZIP › Raw Data/Western Blot/GATA-3/1.png]

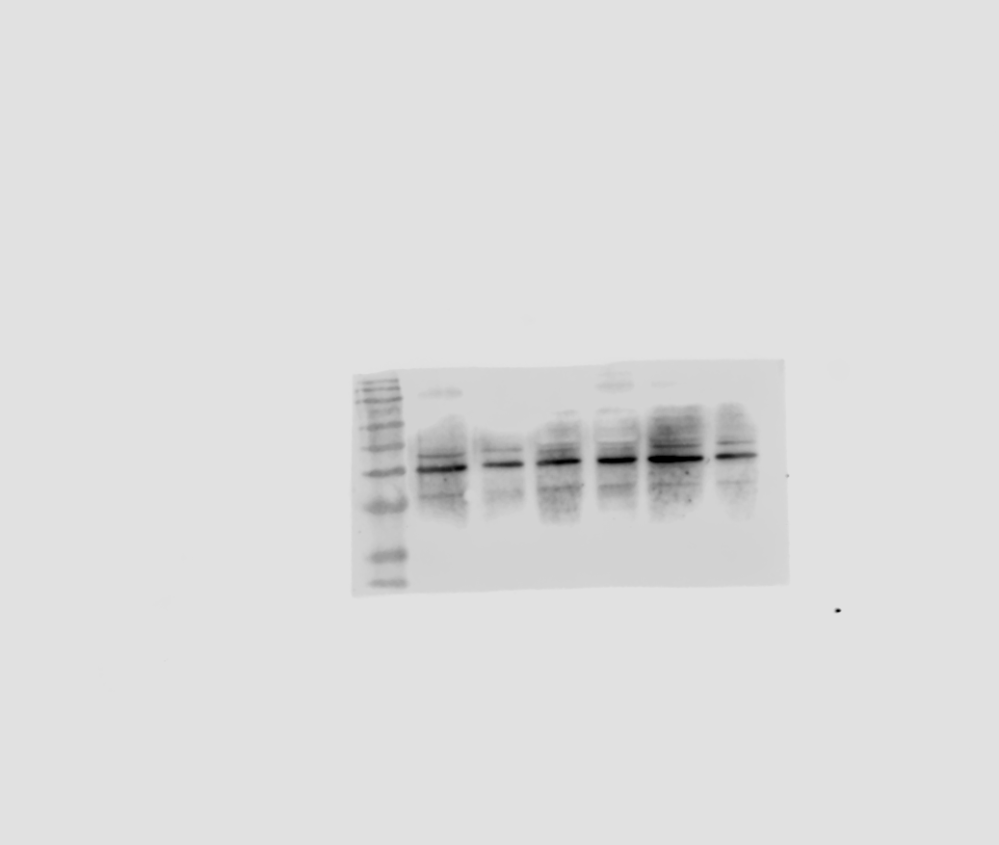

Supplement: Supplementary file 1 [file Data_Sheet_1.ZIP › Raw Data/Western Blot/GATA-3/2.png]

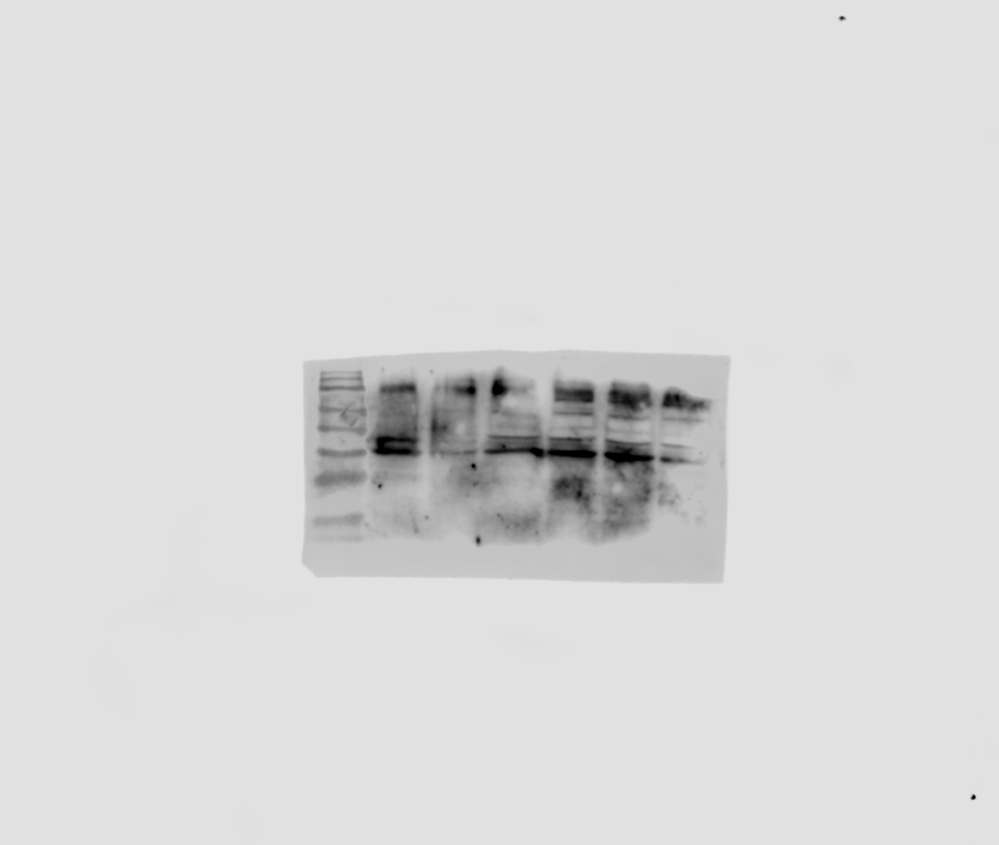

Supplement: Supplementary file 1 [file Data_Sheet_1.ZIP › Raw Data/Western Blot/GATA-3/3.png]

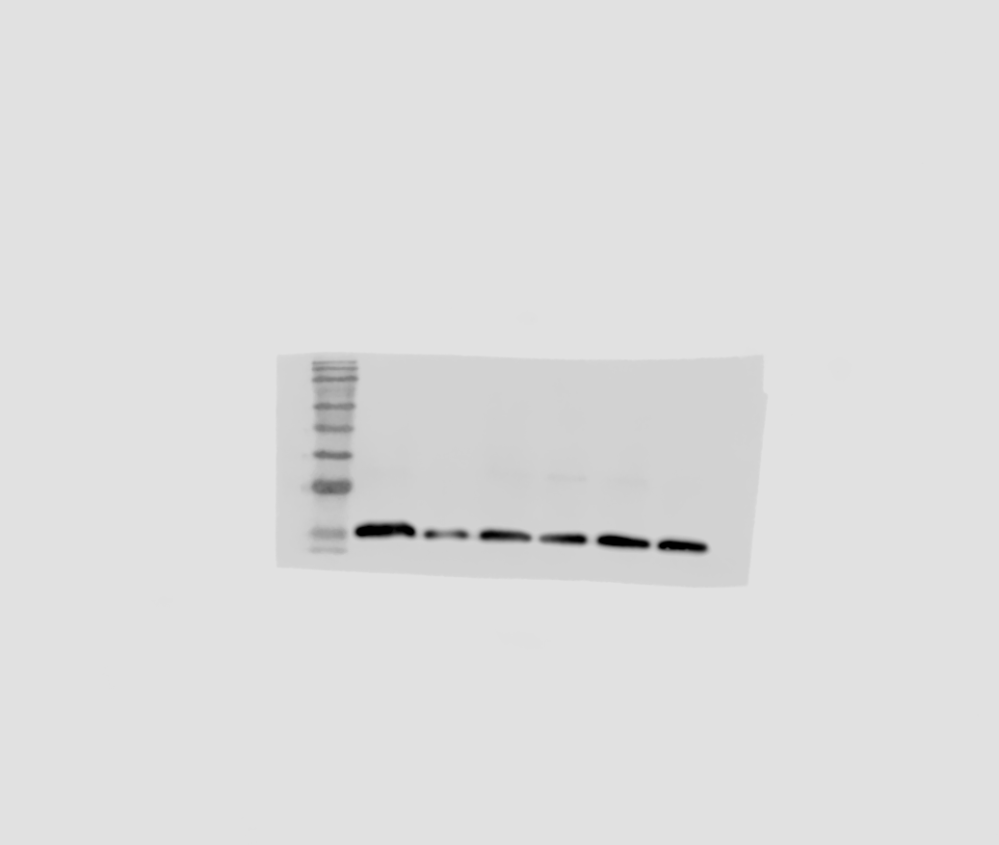

Supplement: Supplementary file 1 [file Data_Sheet_1.ZIP › Raw Data/Western Blot/IFN-γ/1.png]

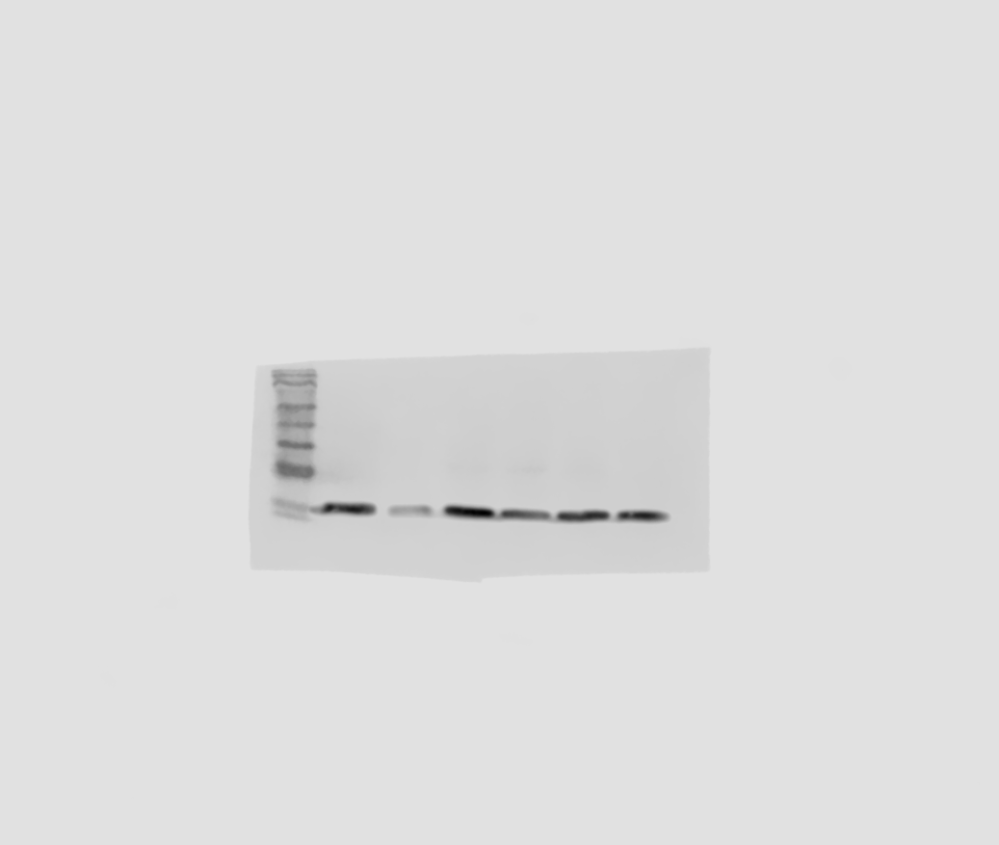

Supplement: Supplementary file 1 [file Data_Sheet_1.ZIP › Raw Data/Western Blot/IFN-γ/2.png]

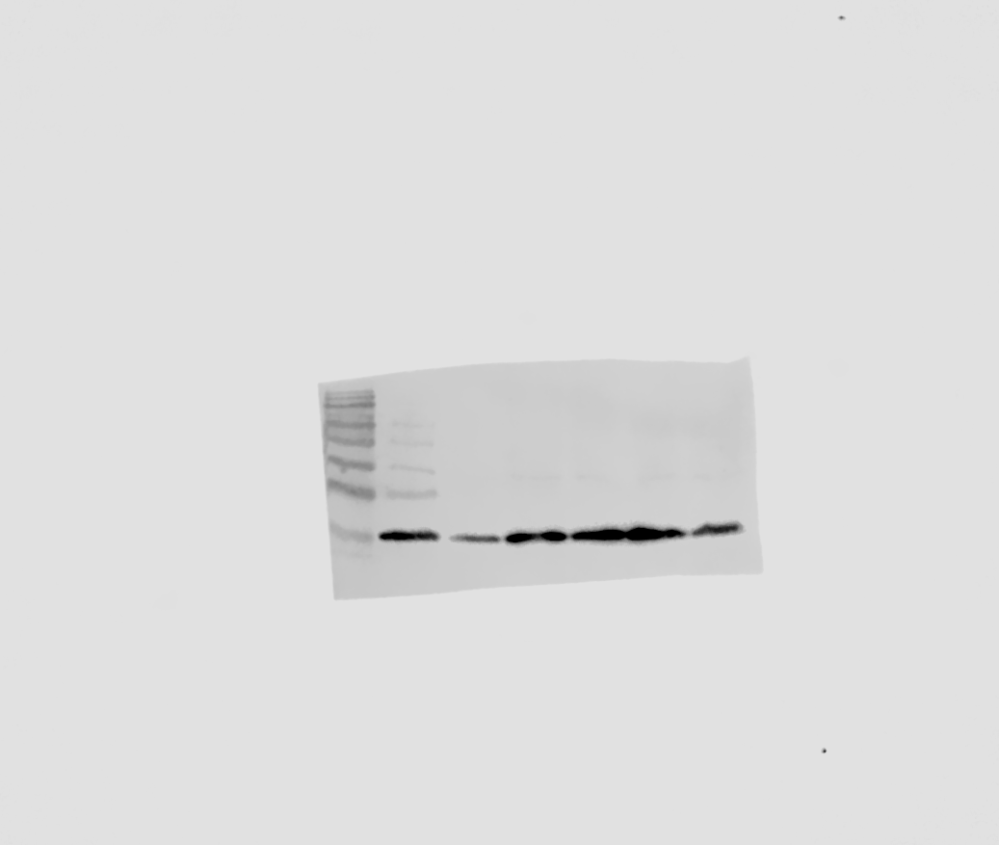

Supplement: Supplementary file 1 [file Data_Sheet_1.ZIP › Raw Data/Western Blot/IFN-γ/3.png]

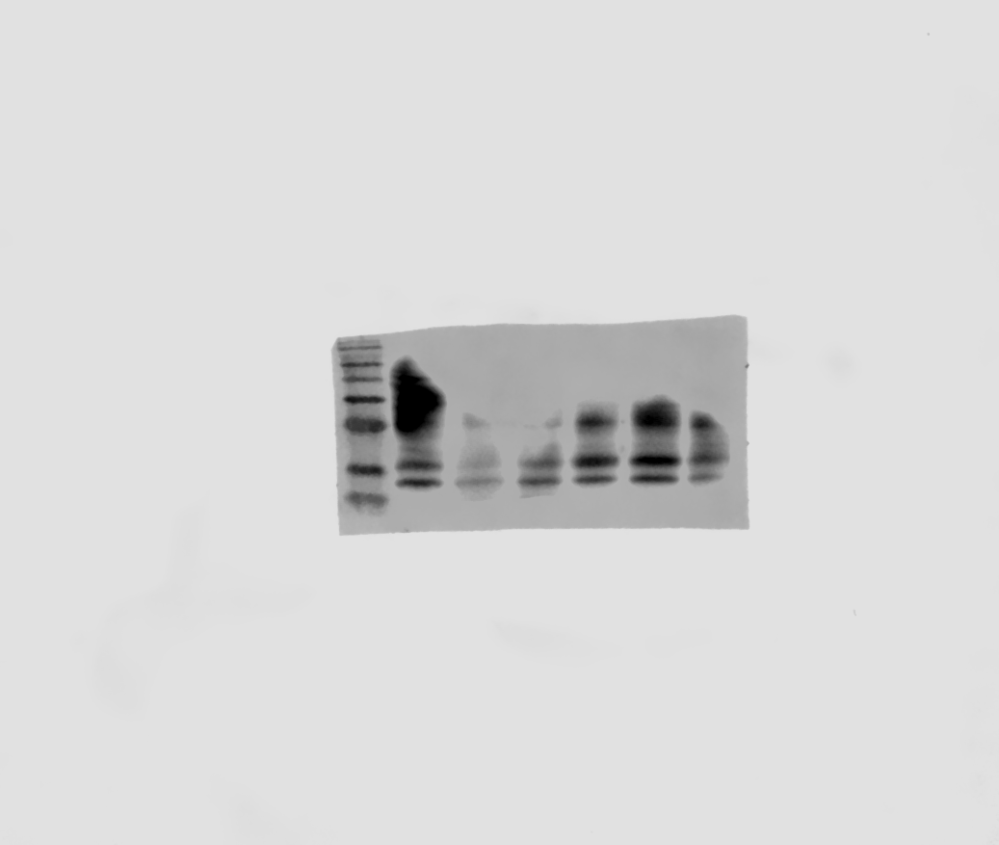

Supplement: Supplementary file 1 [file Data_Sheet_1.ZIP › Raw Data/Western Blot/IL-4/1.png]

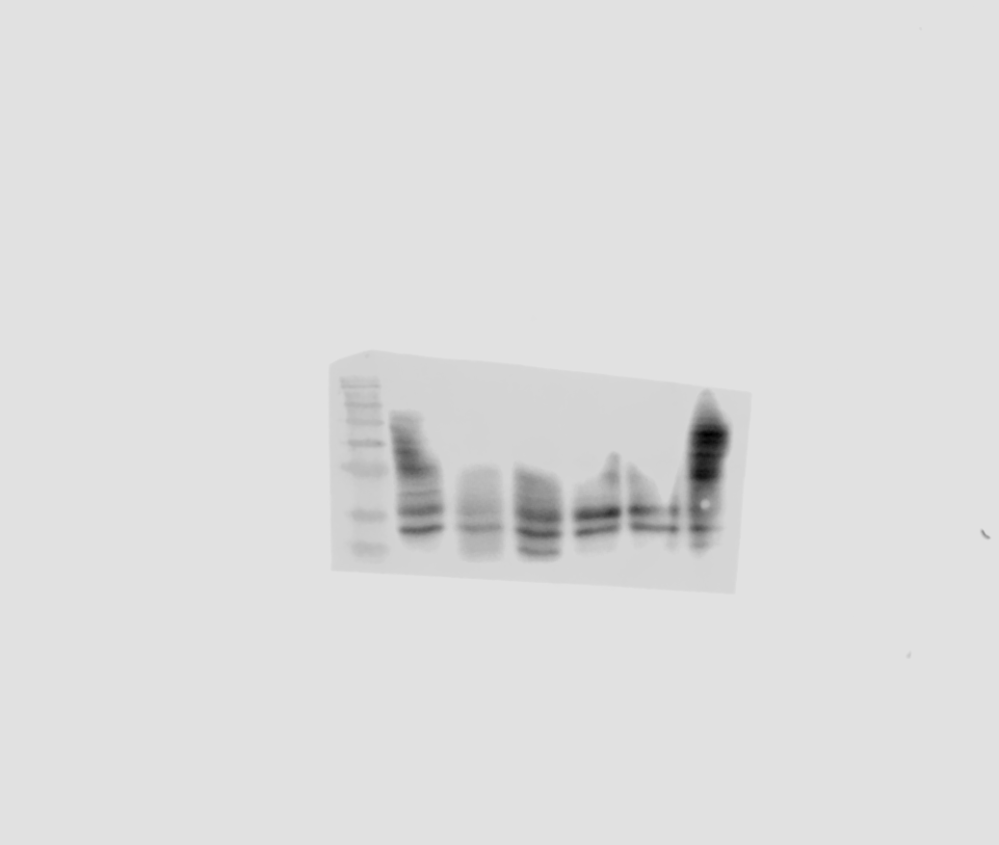

Supplement: Supplementary file 1 [file Data_Sheet_1.ZIP › Raw Data/Western Blot/IL-4/2.png]

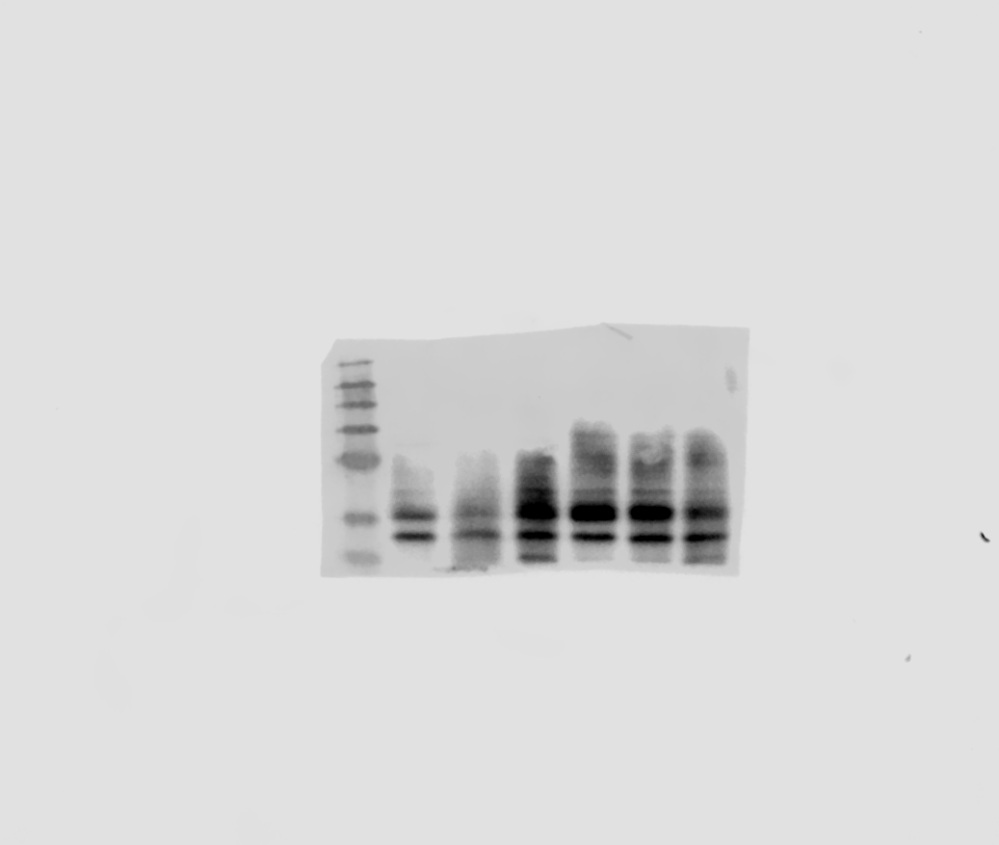

Supplement: Supplementary file 1 [file Data_Sheet_1.ZIP › Raw Data/Western Blot/IL-4/3.png]

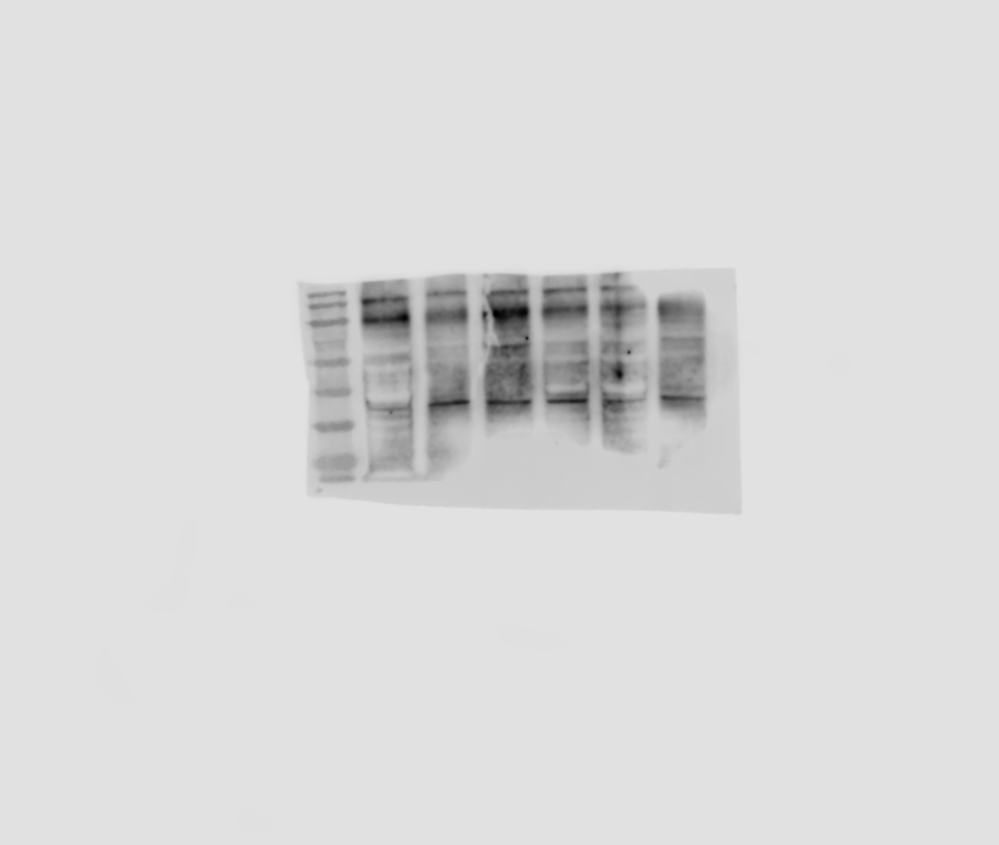

Supplement: Supplementary file 1 [file Data_Sheet_1.ZIP › Raw Data/Western Blot/T-bet/1.png]

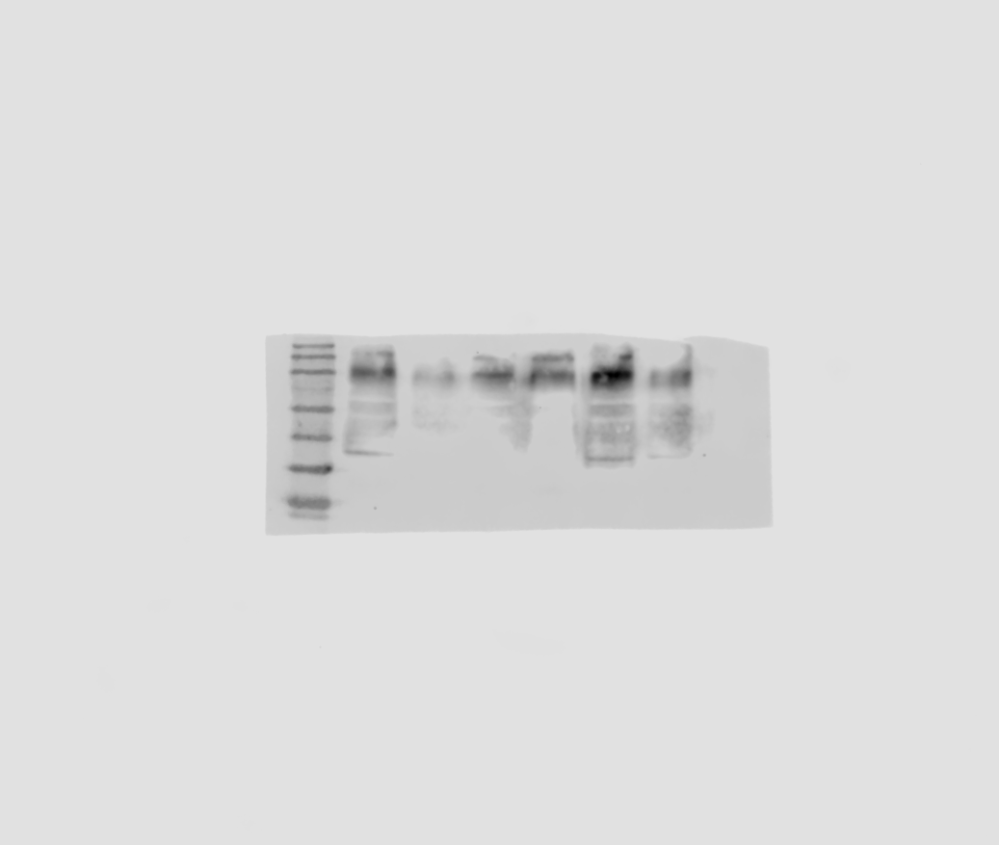

Supplement: Supplementary file 1 [file Data_Sheet_1.ZIP › Raw Data/Western Blot/T-bet/2.png]

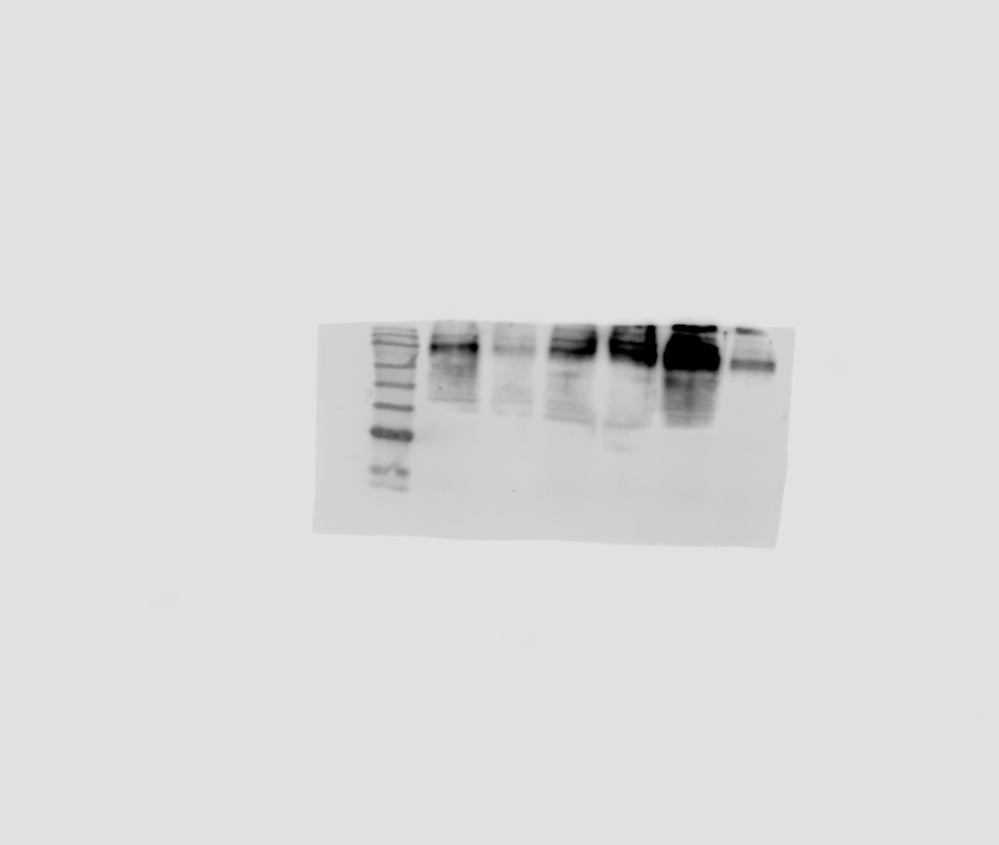

Supplement: Supplementary file 1 [file Data_Sheet_1.ZIP › Raw Data/Western Blot/T-bet/3.png]
